# Crystal structure determination of a lifelong biopersistent asbestos fibre using single-crystal synchrotron X-ray micro-diffraction

**DOI:** 10.1107/S2052252520015079

**Published:** 2021-01-01

**Authors:** Carlotta Giacobbe, Dario Di Giuseppe, Alessandro Zoboli, Magdalena Lassinantti Gualtieri, Paola Bonasoni, Anna Moliterni, Nicola Corriero, Angela Altomare, Jonathan Wright, Alessandro F. Gualtieri

**Affiliations:** a European Synchrotron Radiation Facility, 71 Avenue Des Martyrs, Grenoble, 38040, France; b Department of Chemical and Geological Sciences, University of Modena and Reggio Emilia, Modena, 41121, Italy; c Department of Sciences and Methods for Engineering, University of Modena and Reggio Emilia, Via Amendola 2, Reggio Emilia, 42122, Italy; d Department of Engineering ‘Enzo Ferrari’, Università degli Studi di Modena e Reggio Emilia, Via Università 4, Modena, 41121, Italy; e Pathology Unit, Azienda Unità Sanitaria Locale – IRCCS, Reggio Emilia, Italy; f Institute of Crystallography, CNR, Via Amendola 122/O, Bari, 70126, Italy

**Keywords:** asbestos, micro-diffraction, synchrotrons, fibres, lung diseases, structure determination

## Abstract

The six natural silicates known as asbestos may induce fatal lung diseases *via* inhalation, with a latency period of decades. The five amphibole asbestos species are assumed to be biopersistent in the lungs, and for this reason they are considered much more toxic than serpentine asbestos (chrysotile). Here, we refined the atomic structure of an amosite amphibole asbestos fibre that had remained in a human lung for ∼40 years, in order to verify the stability *in vivo*. The subject was originally exposed to a blend of chrysotile, amosite and crocidolite, which remained in his parietal pleura for ∼40 years. We found a few relicts of chrysotile fibres that were amorphous and magnesium depleted. Amphibole fibres that were recovered were undamaged and suitable for synchrotron X-ray micro-diffraction experiments. Our crystal structure refinement from a recovered amosite fibre demonstrates that the original atomic distribution in the crystal is intact and, consequently, that the atomic structure of amphibole asbestos fibres remains stable in the lungs for a lifetime; during which time they can cause chronic inflammation and other adverse effects that are responsible for carcinogenesis. The amosite fibres are not iron depleted proving that the iron pool for the formation of the asbestos bodies is biological (haemoglobin/plasma derived) and that it does not come from the asbestos fibres themselves.

## Abstract 2

The *in vivo* lifelong stability of an amphibole asbestos fibre extracted from a lung of a patient affected by malignant mesothelioma has been verified using synchrotron X-ray micro-diffraction.

## 1. Introduction   

No one in society is indifferent to the word ‘asbestos’ which refers to a family of six fibrous minerals: the layer silicate chrysotile and the double-chain silicates or amphiboles actinolite asbestos, amosite (cummingtonite–grunerite asbestos), anthophyllite asbestos, crocidolite (riebeckite asbestos) and tremolite asbestos (Case *et al.*, 2011 ▸; Ballirano *et al.*, 2017 ▸).

Chrysotile is a layer silicate with the ideal formula Mg_3_(OH)_4_Si_2_O_5_, composed of one Si-centred tetrahedral (T) sheet joined to one Mg-centred octahedral (O) sheet with a 1:1 (TO) ratio. The TO unit is polar and a misfit exists between the smaller parameters of the T sheet and the larger parameters of the O sheet causing a differential strain between the two sides of the layer. The strain is relieved by rolling the TO layer around the fibril axis to form a cylindrical lattice responsible for the typical fibrous crystal habit of chrysotile (Ballirano *et al.*, 2017 ▸). Amphiboles are double-chain silicates with a Si(Al):O ratio of 4:11. The oxygen atoms of the chains are coordinated not only to Si(Al) but to a variety of other cation sites, yielding the following simplified general formula (Ballirano *et al.*, 2017 ▸) *A* *B*_2_ *C*_5_ *T*_8_O_22_ *W*_2_, with *A* = irregular cation sites (Ca^2+^, K^+^, Li^+^, Na^+^) with coordination in the range 6 to 12; *B* = less regular octahedral or eightfold coordinated cation sites (Ca^2+^, Fe^2+^, Li^+^, Mg^2+^, Mn^2+^, Na^+^), corresponding to the *M*(4) crystallographic site, *C* = regular octahedral cation sites (Al^3+^, Fe^2+^, Fe^3+^, Mg^2+^; Mn^2+^, Mg^3+^, Ti^3+^, Ti^4+^), corresponding to the *M*(1), *M*(2) and *M*(3) crystallographic sites; *T* = tetrahedral sites within the silicate chain (Al^3+^, Si^4+^, Ti^4+^), corresponding to the *T*(1) and *T*(2) crystallographic sites; and *W* = OH^−^, F^−^, Cl^−^,O^2−^. Because of the presence of strong bonds, amphiboles are normally elongated along the crystallographic axis *c* (Ballirano *et al.*, 2017 ▸), and this mono-dimensional character of the structural units (chains) is responsible for their fibrous crystal habit.

Because of its outstanding properties, humans have used asbestos for ∼5000 years in various parts of the world (Gualtieri, 2017 ▸). The massive use of asbestos turned out to be a global problem when animal carcinogenicity tests and long-term epidemiological studies proved that inhalation of asbestos fibres may induce fatal lung diseases like asbestosis, carcinoma, malignant mesothelioma (MM) and many more after a latency period of decades (Manning *et al.*, 2002 ▸). In the mid-50s, Sir Richard Doll for the first time unequivocally reported the link between lung cancer and asbestos fibres inhalation in a cohort of workers exposed to asbestos (Doll, 1955 ▸). Since then, numerous cohort and case control studies have demonstrated the association between asbestos exposure and occupational cancers. In 2012, a working group of the International Agency for Research on Cancer (IARC) published the monograph 100c containing all the epidemiological and carcinogenicity studies conducted from 1970 to that date (IARC, 2012 ▸). Based on the conclusions of the working group, IARC classified all forms of asbestos (*i.e.*, chrysotile and amphibole asbestos) as carcinogenic to humans (Group 1).

Although carcinogenicity of all asbestos minerals is not in question, toxicity potentially is. Because chrysotile and amphibole asbestos have different chemistries, structure models and properties, they also display different biopersistence (Mossman & Gualtieri, 2020 ▸). Biopersistence is the ability of an exogenous particle (such as an asbestos fibre) to persist in the human body regardless of chemical dissolution (biodurability) and physical clearance mechanisms (Bernstein *et al.*, 2005 ▸). Because *in vitro* (biodurability) and *in vivo* (biopersistence) animal and human studies have shown that chrysotile is much less biodurable/biopersistent (Bernstein *et al.*, 2005 ▸; IARC, 2012 ▸; Utembe *et al.*, 2015 ▸) than amphibole asbestos in the body, the toxic potential of chrysotile is assumed to be low with respect to that of amphibole asbestos (Mossman, 1993 ▸). It was demonstrated that during the process of phagocytosis by alveolar macrophages (AMs) *in vivo* the acid environment produced intracellularly attacks chrysotile fibres that rapidly undergo a process of dissolution, with leaching of Mg and production of amorphous silica relicts (Gualtieri *et al.*, 2019 ▸) easily eliminated by macrophages (Bernstein *et al.*, 2013 ▸). Oppositely, amphibole asbestos fibres are biodurable (Bernstein *et al.*, 2013 ▸) and are prone to induce chronic inflammation responsible for adverse effects *in vivo*. Based on this model, only the amphibole asbestos species are banned worldwide and 65% of the countries in the world (including China, India and Russia) still mine and allow a ‘safe use’ of chrysotile asbestos (Gualtieri, 2017 ▸). The large body of evidence supporting this model based on biopersistence relies on microscopic (mostly electron microscopy) observations of the fibres and determination of their overall crystallinity (Wagner *et al.*, 1974 ▸; Pooley, 1976 ▸; Langer & Nolan, 1994 ▸; Muhle *et al.*, 1994 ▸).

Besides the morphological and chemical characterization conveyed by ESD-supported electron microscopy (Gordon, 2019 ▸; Gandolfi *et al.*, 2016 ▸), X-ray diffraction is considered a reliable tool for the characterization of asbestos fibres. This technique has recently been used for the direct analysis of fibres found in tissues of rats subjected to intraperitoneal/intrapleural injection of Union for International Cancer Control (UICC) chrysotile, UICC crocidolite and erionite–Na (Gualtieri, 2017 ▸). Powder-diffraction data were collected from bundles of fibres and this gave an accurate picture that was averaged over several crystallites. Owing to problems of peak overlap in powder data, it is not possible to carry out a free refinement of these complex atomic structures in order to detect and measure subtle chemical changes that we need to detect to prove the biopersistence. Then, in order to see details in the atomic structure, single crystal X-ray diffraction is the ideal technique, if the problem of tiny crystallite sizes can be overcome. Advances in synchrotron beamlines bring opportunities with accompanying challenges for the study of important structural parameters such as atomic coordinates and site population.

The ID11 beamline at the European Synchrotron Radiation Facility (ESRF) has a strong tradition of offering single-crystal diffraction data at high energy/short wavelength (for example, at an energy of 40 keV or a wavelength of ∼0.3 Å). One of the key advantages of using a short wavelength is the increase in data resolution and the possibility of investigating further out into reciprocal space and reducing the size of the blind region on the rotation axis (Wright *et al.*, 2020 ▸).

Since 2016, ID11 has offered a new endstation called ‘nanoscope’ where it is possible to focus the beam to the deep sub-micrometre scale. This is possible by using in-line crossed silicon compound refractive lenses (Snigirev *et al.*, 2007 ▸) and using a crossed pair of vertical and horizontal line foci. Combining the very small beam size with a diffractometer to align and maintain a sample in the beam during rotation is very promising for the crystallographic characterization of natural fibres. Recently, it was shown how using such a small beam size to study natural fibres can lead to precise and unambiguous structure solution and refinement (Giacobbe *et al.*, 2018 ▸, 2019 ▸).

Here we report a proof of the *in vivo* biopersistence of asbestos fibres in human lung tissues at the atomic scale using synchrotron micro-diffraction. We show that the atomic structure of an amosite fibre remained stable for ∼40 years in the lungs of a subject diagnosed with MM and originally exposed to a mixture of chrysotile, amosite and crocidolite.

## 2. Experimental   

### 2.1. Description of the subject   

Lung samples were obtained during the autopsy of a male subject who was diagnosed with MM and died in 2017. MM was localized in the parietal pleura of the left lung with extensive involvement of pulmonary parenchyma, surrounding thoracic soft tissues; the eighth, ninth and tenth ribs; the pericardium; the apex of the heart; and the ascending aorta. The patient worked for ten years (from 1970 to 1980) in a factory producing asbestos-containing products situated in a manufacture district of Northern Italy. Before the ban of all asbestos minerals in Italy in 1992, the company used asbestos fibres for the production of cement products, such as pipes, flues, rainwater systems, tanks and profiled sheeting/corrugated roofs. In the mixtures for fibre–cement products, chrysotile represented the most widely used asbestos variety together with crocidolite, until 1986 when the use of crocidolite was banned in the countries of the European Community including Italy. Until the early 1970s, the use of amosite was also reported for the preparation of cement–asbestos mixtures (Viani *et al.*, 2013 ▸). During his work, the subject was exposed to three asbestos species: chrysotile, crocidolite and amosite. From the history data of the subject, we hypothesize that the residence time of the fibres in the lung was 37–47 years. The first evidence of the neoplasm was found a year before his death. The patient underwent computed-tomography scans showing a diffuse pleural thickening compatible with MM in the left lung.

### 2.2. Separation of the mineral fibres from the lung tissues   

Portions of tumour-free parenchyma of the left lung, previously stored in formaldehyde solution buffered at neutral pH, were selected for extracting mineral fibres from the tissue. To obtain mineral fibres from the lung tissues, the organic matter was oxidized using a low-temperature plasma asher (LTA). For the LTA procedure, lung samples were freeze dried using a lyophilizer. Approximately 10 mg of freeze-dried lung was ashed with an Emitech K1050X plasma asher (Ashford, UK). LTA was carried out at 80 °C, with an energy input of 100 W and an oxygen flow of 225 ml min^−1^. After 20 h, the ash was suspended in a solution made of 50 ml distilled water and 2 ml ethyl alcohol, and filtered using a polycarbonate 0.2 µm pore-size filter.

### 2.3. FEG-SEM   

Scanning electron microscopy (SEM) analyses were performed using an FEI Nova NanoSEM 450 FEG-SEM (field emission gun-SEM) with 15 kV accelerating voltage and 3.5 µA beam current. Portions of the polycarbonate filters were coated in gold (10 nm of thickness), using a gold sputter coater (Emitech K550), and placed on an aluminium stub with double-stick carbon tape. The chemical composition of fibre specimens was investigated by energy-dispersive X-ray spectroscopy (EDS) and carried out by using an X-EDS Bruker QUANTAX-200. The shape and size parameters of the amphibole asbestos fibres were determined on ∼100 individual fibres using high-magnification (>×40000) FEG-SEM images.

### 2.4. TEM and STEM   

Transmission electron microscopy (TEM) investigations were carried out using a Talos F200S G2 microscope (Thermo Fisher Scientific, Waltham, USA), equipped with an S-FEG Schottky field emitter operating at 200 kV and two large-area energy-dispersive X-ray (EDX) spectrometers with silicon drift detectors. The investigated samples were suspended with 1 ml of acetone in a test tube, sonicated for 20 min (using a low-power sonic bath) and left to set for 5 min. Then, a drop of the suspension was transferred onto a 300-mesh carbon copper TEM grid (Ted Pella Inc., Redding, USA) and left to dry. Observations were made both in TEM and scanning TEM (STEM) mode.

### 2.5. Synchrotron micro-diffraction   

Single-crystal micro-diffraction data were collected at ID11, the materials science beamline of the ESRF. The studied fibres were selected from a polycarbonate 0.2 µm pore-size filter containing the ash resulting from the lung dissolution procedure described above and mounted onto MiTeGen Microloops (see the Supporting information, Fig. S1). All the data were collected at the ‘nanoscope’ station, where the sample is rotated on a high-precision air-bearing axis (Leuven Air Bearing RT120UP). Centring of the crystal on the rotation axis is achieved using a NanoPos hexapod (Symétrie) mounted on a piezoelectric *xyz* stage, where the minimum incremental movement is 10 nm in the vertical and in the two horizontal directions. The sample tower, rotation and axis positioning collectively have a mechanical performance that is ∼40 nm at the sample position.

The beam size (500 nm in the vertical dimension × 800 nm in the horizontal dimension) was measured by placing a small wire cross on the rotation axis for alignment purposes (Goodfellow W005200, 5 µm diameter tungsten wire, plated with gold coating that is 3–5% in weight). This cross gives strong fluorescence signals enabling one to carry out ‘knife-edge scans’, and the individual crystallites of gold in the surface plating give strong and sharp diffraction signals (Fig. S2). Prior to the collection of diffraction data, the crystal size was measured by scanning the fibre across the 500 × 800 nm beam and analysing the standard deviation signal on the diffraction detector. Fig. S3 shows fits for the *x* (*a*) and *y* (*b*) dimensions of the amphibole giving values of 1.12 and 1.08 µm, respectively. Diffraction images were recorded using a wavelength of 0.3091 Å, obtained *via* a bent Si (111) Laue–Laue double-crystal monochromator (relative bandwidth Δλ/λ ≃ 10^−3^). A Frelon4M detector (ESRF, Grenoble, France) with 2048 × 2048 pixels of 50 × 50 µm was used with a sample-to-detector distance of 125.48 mm. Rotation scans were recorded over an angular range of 360° about the vertical *z* axis in steps of 0.25°. Detector distance and tilts were calibrated using a CeO_2_ powder standard.

A 30 µm long amosite fibre was analysed. Two sets of data were measured focusing the X-ray beam at different zones of the fibre, *i.e*. at the top edge (fibre_T) and at the centre (fibre_C), with the aim of investigating the occurrence of structural changes in the fibre. The .*edf* diffraction images were recorded by a Frelon4M detector and were converted into a standard Esperanto format using the open-source software script *FREAC* (Dyadkin, https://soft.snbl.eu/freac.html) before processing them in the *CrysAlis* software (Agilent, 2013 ▸). Bragg peaks were indexed and their intensities were integrated and corrected for Lorentz polarization effects, using the *CrysAlis^Pro^* package (Agilent, 2013 ▸). Absorption effects were corrected using *SCALE3 ABSPACK* of *CrysAlis* (Oxford Diffraction, 2006 ▸) *via* a multi-scan semi-empirical approach. *R*_int_ values of 3.0% for fibre_T and 4.7% for fibre_C were obtained (with a data resolution of 0.75 Å), see Table 1 ▸.

The crystal structure was solved by direct methods using *SIR2019* (Burla *et al.*, 2015 ▸) and refined by a full-matrix least-squares technique on *F* ^2^ by *SHELXL-2014* (Sheldrick, 2015 ▸), where *F* is the structure factor modulus. The absorption coefficient and real and imaginary dispersion coefficients of each atomic element were calculated by *VESTA* (Momma & Izumi, 2011 ▸) at the experimental wavelength and supplied to *SHELXL-2014*. *WinGX* (Farrugia, 2012 ▸) and *publCIF* (Westrip, 2010 ▸) were used for preparing the material for publication. The main details on crystal data, data collection and structure refinement for fibre_T and fibre_C are provided in Table 1 ▸.

## 3. Results   

### 3.1. SEM   

**Figure 1:**
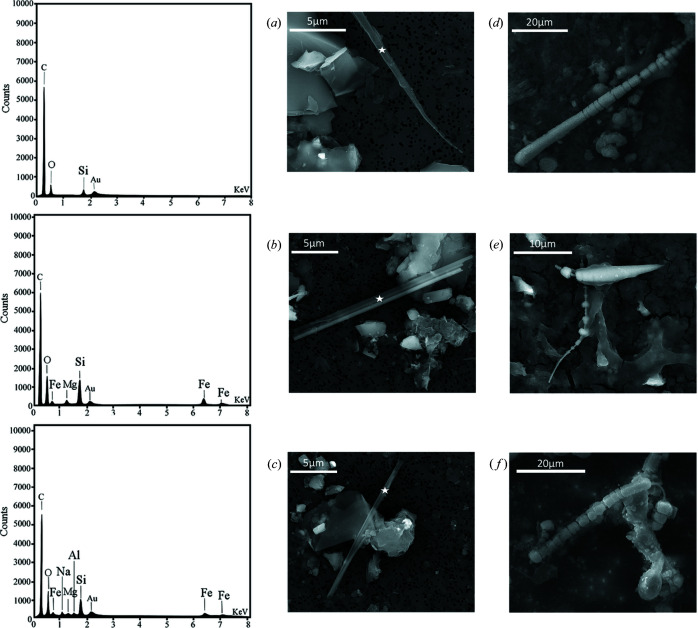
A gallery of acquired FEG-SEM pictures and EDX point spectra of uncoated and coated fibres found in the lung tissues of the male subject. (*a*) The metastable product of the dissolution of chrysotile. The EDX spot analysis (shown at the top left) indicates that the fibre is only composed of SiO_2_. (*b*) Amosite fibre with the corresponding EDX spectrum on the left showing the bands assigned to the three more abundant elements of amosite, *i.e.* Mg, Si and Fe. (*c*) Crocidolite fibre with the corresponding EDX spectrum (at the bottom left) showing the presence of Na in addition to Fe, Si and Al. Au is from the sample coating. The observed ABs exhibit multiform shapes. Forms: (*d*) lancet-like, (*e*) beaded/dagger-like and (*f*) beaded/cylindrical-like shapes.

**Figure 2:**
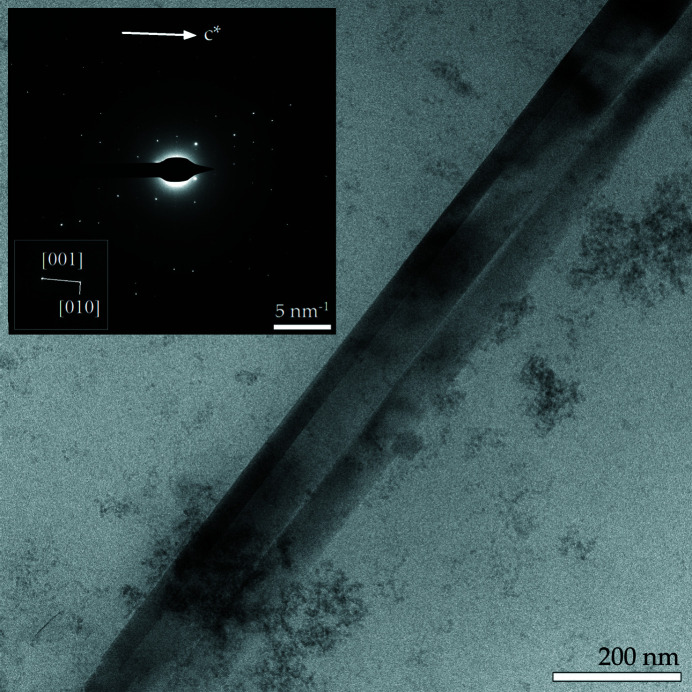
A TEM image of a highly crystalline amosite fibre after long permanence in the patient’s lung. At the top left, a SAED pattern of the core of the fibre is shown. SAED shows the typical diffraction pattern of a crystalline material. The *d* spacings (Å) and the angle determined from the pattern are: 18.3 Å along [010], 5.3 Å along [001] and α = 90°. The calculated cell parameters are consistent with the data reported by Pollastri *et al.* (2017 ▸) and Fischer (1966 ▸).

**Figure 3:**
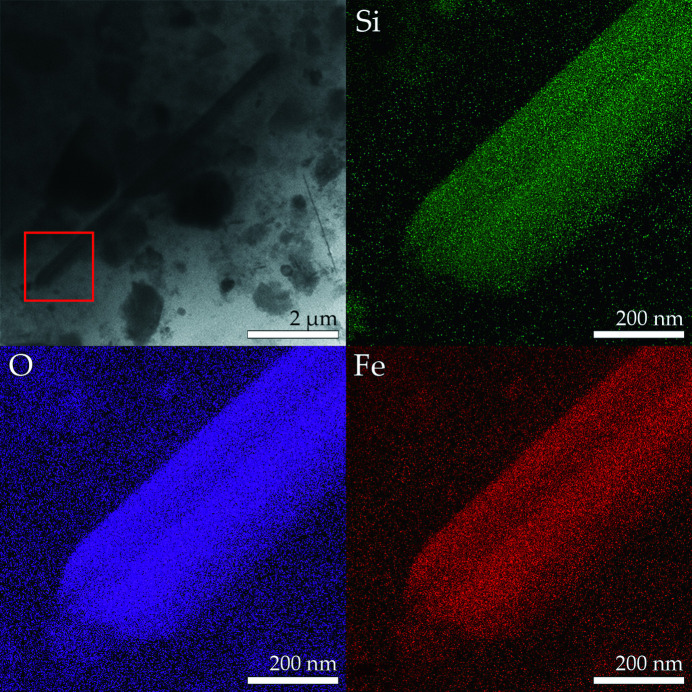
A STEM image of an uncoated amosite fibre with the chemical mapping showing its characteristic chemical elements (Si, Fe and O) with the corresponding STEM-EDX maps. The red square indicates the area selected to carry out the EDX mapping.

The solid fraction obtained from the ashing and dissolution of the lung tissues of the human subject contained both free fibres and fibres coated with asbestos bodies (ABs) (Fig. 1 ▸). Identification of the nature of fibres based upon EDX micro-analysis and selected-area electron diffraction (SAED) patterns (Figs. 1 ▸ and 2 ▸, respectively) showed that 97 (2)% of the fibres detected in the solid suspension were crocidolite [Mg/Si/Fe: Fig. 1 ▸(*c*)] and amosite [Al/Si/Fe: Fig. 1 ▸(*b*)], and the remaining 3(1)% were silica-rich fibres, *i.e*. the metastable product from dissolution of chrysotile owing to loss of magnesium by leaching [Fig. 1 ▸(*a*)]. Both the SEM and STEM investigations did not reveal any crystalline chrysotile (Mg/Si) fibres, showing that after ∼40 years of residence time in the lungs of the subject the major source of exposure at the workplace (85 wt% chrysotile fibres) was largely dissolved or cleared. Free amphibole asbestos displays a wide range of lengths (*L*), with values ranging from 8.69 to 51.0 µm, but the longer fibres predominate in the population. In fact, more than 75% of the fibres were longer than 19.1 µm and 50% were longer than 22.3 µm, confirming that biodurable fibres with *L* > 10 µm are too long to be eliminated by AMs (generally assumed to have a size of ∼10 µm). The widths (*W*) of the fibres ranged overall between 0.22 and 1.23 µm, with 50% of the fibres showing a width of <1.00 µm. According to the World Health Organization guidelines (World Health Organization, 1997 ▸), fibres with respirable size are those equal to or longer than 5 µm with diameters up to 3 µm and an aspect ratio of *L*/*W* ≥ 3. All mineral fibres recovered from the lung tissue of the human subject fit the World Health Organization criteria. The observed ABs display a large variety of forms including lancet, beaded-dagger and beaded/cylindrical-like shapes [Figs. 1 ▸(*d*), 1 ▸(*e*) and 1 ▸(*f*)]. SEM observations showed that segmented ABs are regularly spaced along the fibre [Fig. 1 ▸(*f*)] but sometimes vary in diameter from one end of the fibre to the other [Figs. 1 ▸(*d*) and 1 ▸(*e*)]. These results are in line with those reported by Di Giuseppe *et al.* (2019 ▸) who investigated the process of nucleation and development of ABs on the surface of fibres inside lung tissues. ABs only develop around biodurable fibres that are longer than the mean diameter of AMs and thus cannot be engulfed, prompting frustrated phagocytosis. AB encapsulation occurs as a last defence mechanism of the body aimed at isolating the fibre from the extracellular environment. As an example, the TEM micrograph and SAED pattern in Fig. 2 ▸ show that the amphibole asbestos fibres recovered from the lung tissues were crystalline even after long times inside the tissues. From Fig. 2 ▸, the *d* spacings return a value of 18.3 Å along [010] and 5.3 Å along [001]. These values are consistent with the *d* spacings of the amosite sample studied by Pollastri *et al.* (2017 ▸) and Fischer (1966 ▸). Fig. 3 ▸ is an example STEM image of an uncoated amosite fibre and relative EDX chemical mapping showing its characteristic chemical elements (Si, O and Fe). Spatial distribution of the selected elements appears uniform along the fibre and no signs of enrichment or depletion of the elements on the edges or core of the fibre were found.

### 3.2. Diffraction   

From the micro-diffraction data collected at the nanoscope station of ID11 and the chemical results obtained from the EDX analysis, the fibre could be identified as amosite, belonging to the cummingtonite–grunerite solid-solution series as the Mg/(Mg + Fe) mole-fraction value is 0.17 [*X*(Mg) > 0.3 for cummingtonite and *X*(Mg) < 0.3 for grunerite, where *X*(Mg) is the weight percentage of Mg] (Leake, 1978 ▸). It crystallizes in the monoclinic system, space group *C*2/*m*. In the following, we report the results for the two data sets collected on the top of the fibre (fibre_T) and on the centre of the fibre (fibre_C). The cell parameters and volume in the cases of fibre_T and fibre_C are *a* = 9.5440 (2) Å, *b* = 18.2455 (4) Å, *c* = 5.32660 (12) Å, β = 101.883 (2)° and *V* = 907.67 (3) Å^3^, and *a* = 9.5343 (2) Å, *b* = 18.22210 (4) Å, *c* = 5.3202 (2) Å, β = 101.846 (3)° and *V* = 904.57 (4) Å^3^, respectively, and are in agreement with the model proposed by Finger (1969 ▸).

For both fibre_T and fibre_C cases, the structure solution step was carried out by assuming that the four *M*(1)…*M*(4) sites were fully occupied by Fe atoms and that the *T*(1) and *T*(2) sites were fully occupied by Si atoms. During the structure refinement performed by *SHELXL-2014*, the substitutional disorder, typical of these kinds of compounds, was accounted for by applying the following restraints and constraints (Müller *et al.*, 2006 ▸).

(1) Free variables linked to the site-occupancy factors of groups of disordered atoms were refined assuming that their fractions had to sum up to the target value (*i.e*. the site occupancy). As a starting point, it was assumed that *T* sites were shared by Si and Al atoms, while *M* sites were shared by Fe atoms and one (or more than one) additional cation(s) (*i.e*. Mg and/or Al and/or Ca atoms).

(2) Disordered atoms sharing a crystallographic site were constrained to have both the same crystallographic coordinates and the same anisotropic displacement parameters.

A careful inspection of the electron-density map calculated by difference Fourier synthesis aimed at positioning a hydrogen atom bonded to the O(3) atom located at the *W* site enabled us to detect, among the candidate peaks, the most promising position in terms of bond distance and angle; it has also been investigated if the *W* site was shared by an O atom and an additional anion (*i.e*. an F^−^ or a Cl^−^ atom).

The results of the refinement indicate that for fibre_T the anion sharing the *W* site is F^−^ instead of Cl^−^ (for fibre_C, a negative refined value of the corresponding free variable linked to the site-occupancy factor was obtained).

**Figure 4:**
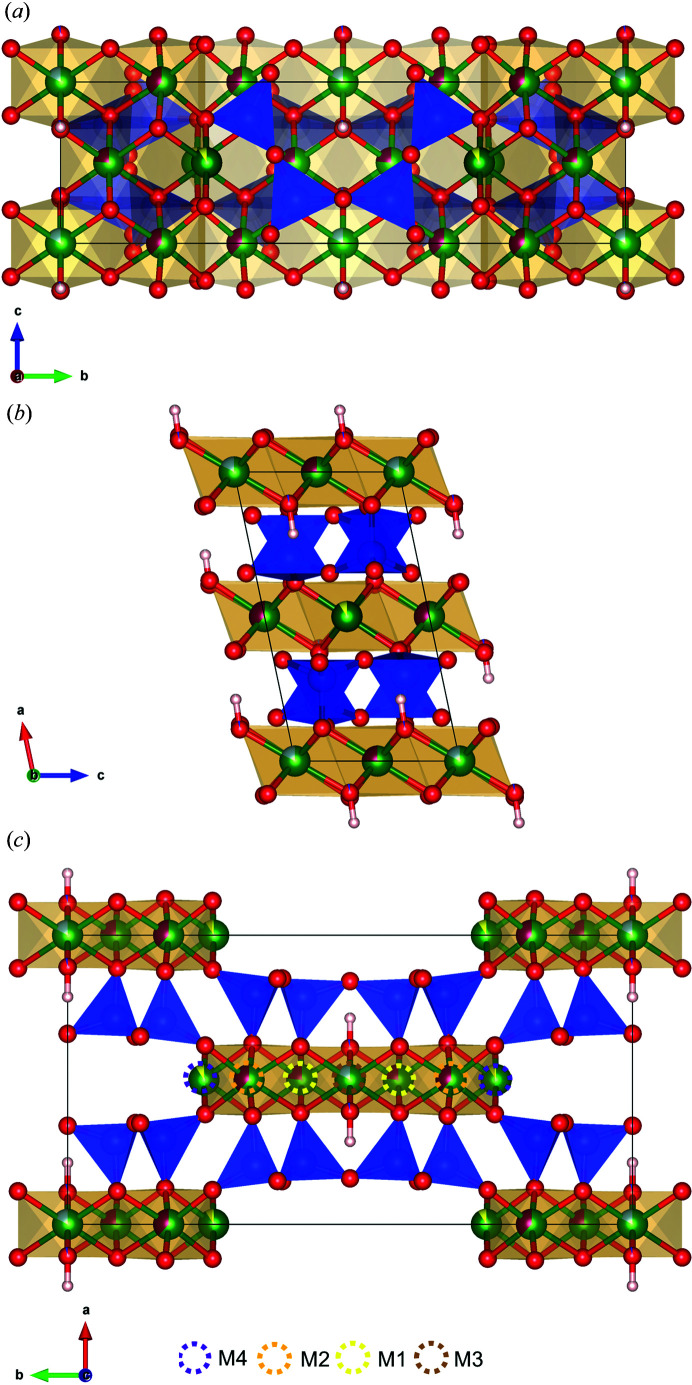
The structure model of fibrous amosite (fibre_T). (*a*) Projection along *a*, (*b*) projection along *b* and (*c*) projection along *c*. Legend: blue polyhedra = tetrahedral centred by Si, blue balls = Si atoms, green balls = F atoms, red balls = O atoms, purple balls = Mg atoms, yellow balls = Ca atoms, light blue balls = F substituting O atoms. The dotted line circles are used to better illustrate the amphibole sites (Hawthorne, 1983 ▸). The plots were created using the *VESTA* software (Momma & Izumi, 2011 ▸).

In the case of fibre_T, a view along *a, b* and *c* of the crystal packing is given in Fig. 4 ▸ (for fibre_C, refer to Fig. S4). For fibre_T, the tetrahedral bond lengths *T*(1)–O range from 1.610 (4) Å to 1.627 (4) Å while the tetrahedral bond lengths *T*(2)–O range from 1.604 (4) Å to 1.647 (4) Å (Table S1). One could note that the *T*1–O tetrahedron is more regular than the *T*2–O tetrahedron (Δ = 0.146 versus 0.955, see Table 2 ▸), as is confirmed by O–*T*1–O bond angles compared with O–*T*2–O bond angles (see later in the text). Similar results were achieved for fibre_C (see Tables S2 and 2 ▸).

The mean bond lengths *M*–O of the sites in the octahedral strip of the refined data sets are in general agreement with the trends first outlined for *C*2*/m* amphiboles in the work of Hawthorne (1983 ▸).

The *M*(l), *M*(2), *M*(3) and *M*(4) sites for fibre_T and fibre_C are characterized by similar mean *M*–O bond lengths: 2.104 (4) Å [*M*(1)–O], 2.113 (4) Å [*M*(2)–O], 2.096 (5) Å [*M*(3)–O] and 2.275 (4) Å [*M*(4)–O] for fibre_T, and 2.101 (4) Å [*M*(1)–O], 2.112 (4) Å [*M*(2)–O], 2.095 (5) Å [*M*(3)–O] and 2.272 (4) Å [*M*(4)–O] for fibre_C.

The metal distribution over the *M* sites can be described as follows: *M*(1) = 0.763 (13) Fe^2+^ and 0.237 (13) Mg for fibre_T and *M*(1) = 0.744 (13) Fe^2+^ and 0.256 (13) Mg for fibre_C, *M*(2) = 0.615 (12) Fe^2+^ and 0.385 (13) Mg for fibre_T and *M*(2) = 0.600 (13) Fe^2+^ and 0.400 (13) Mg for fibre_C, *M*(3) = 0.827 Fe^3+^ and 0.173 Al^3+^ for fibre_T and *M*(3) = 0.785 (19) Fe^3+^ and 0.215 (19) Al^3+^ for fibre_C, and *M*(4) = 0.908 (19) Fe^2+^ and 0.092 (19) Ca^2+^ for fibre_T and *M*(4) = 0.914 (19) Fe^2+^ and 0.086 (19) Ca^2+^ for fibre_C.

Usually the oxidation states of iron, their distribution and their relative fraction in the structure of amphibole asbestos species are determined with the aid of Mössbauer spectroscopy (see, for example, Pollastri *et al.*, 2015 ▸). In this case, the application of this bulk technique to a single fibre was not possible and the distribution of iron relied only upon the results of the structure refinement.

The iron distribution in the amosite fibre structure follows the site preference expected for iron–magnesium–manganese amphiboles, namely, *M*(4) > *M*(3) > *M*(1) > *M*(2) (Hawthorne, 1983 ▸).

The *M*(4) position accommodates the larger ions, namely Ca^2+^ and Fe^2+^, where one could note a different proportion in concentration of these two cations in the two different parts of the fibre.

**Figure 5:**
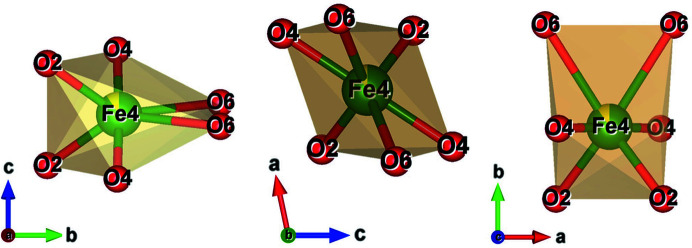
The distorted configuration for *M*(4) octahedra. The *M*(4)–O bond distances are: *M*(4)–O(2) = 2.147 (4) Å, *M*(4)–O(4) = 2.004 (4) Å and *M*(4)–O(6) = 2.66 (4) Å. The plots were generated using the *VESTA* software (Momma & Izumi, 2011 ▸).

However, as already shown in similar amphiboles structures [*e.g.*, cummingtonite described in the work of Ghose (1961 ▸)], the *M*(4) polyhedra are highly distorted (see Fig. 5 ▸). For fibre_T the *M*(4)–O bond distances are: *M*(4)–O(2) = 2.147 (4) Å, *M*(4)–O(4) = 2.004 (4) Å and *M*(4)–O(6) = 2.674 (4) Å (very similar values can be found in Table 3 ▸ for fibre_C).

The short *M*(4)–O(4) bond suggests that there might be covalent bonding between these two oxygens and *M*(4), which is mostly occupied by Fe^2+^. These covalent bonds are probably stabilizing the structure and favouring this type of Mg–Fe ordering, as already seen for the cummingtonite.

The short *M*(4)–O(4) bond indicates that the linkage between *M*(4) and O(4) is stronger than a single *M*–O bond because, while oxygen O(1) and O(2) are bonded to one silicon and three metals each, O(4) is bonded to one silicon and two metals.

Though the Si–O distances within two *T*–O tetrahedra are not significantly different, the O–*T*–O bond angles show that the *T*(1)–O tetrahedron is more regular, in which O–*T*(1)–O bond angles range for fibre_T (see Table S1) from 108.5° (3) [O(7)–Si(1)–O(6)] to 110.9° (2) [O(7)–Si(1)–O(1)], while O–Si(2)–O bond angles range from 102.1° (2) [O(6)–Si(2)–O(4)] to 115.9° (2) [O(4)–Si(2)–O(2)]. Similar values are observed in the case of fibre_C (see Table S2). It should be noted that the two distorted tetrahedral angles in the Si(2)–O tetrahedron involve O(4). Hence, the distortion in the *T*(2)–O tetrahedron is certainly caused by the strong attraction between O(4) and *M*(4).

Generally, there are no significant variations among the electron occupancies for fibre_T and fibre_C. Their structure models are strongly overlapping, with an r.m.s. deviation of 0.016 (the largest distance between correspondent atoms is 0.296 Å and involves the hydrogen atoms, the rest of the atomic positions of the two structure models are very close), and are characterized by similar site partitions of the sharing-site atoms, except for the F content in the *W* site. Fibre_T, especially, is affected by the substitution of OH^−^ by F^−^, possible in Ca^2+^ amphiboles (Ekström, 1972 ▸), with a site partition of 0.96 (3) for O and 0.04 (3) for F.

The coordinates of the hydrogen site obtained in this study are consistent with previous experimental findings (Ghose, 1961 ▸). Although a PLAT420_ALERT_2_B alert is generated during the CIF validation process *via publCIF* (Westrip, 2010 ▸), as no suitable acceptors satisfying the commonly used hydrogen-bond criteria (Jeffrey, 2003 ▸) are found, we consider that there are three potential weak hydrogen bonds with O(6) × 2 and O(7) as acceptors [*i.e*. H⋯O(6) = 2.975 Å, O(3)⋯O(6) = 3.402 Å and O(3)–H⋯O(6) = 111.75°; and H⋯O(7) = 3.177 Å, O(3)⋯O(7) = 3.496 Å and O(3)–H⋯O(7) = 103.9°, see Fig. S5]. Indeed, the PLAT420_ALERT_2_B occurs because the possible hydrogen bonds cannot be classified. They are neither strong nor moderate but satisfy the range values of the H⋯A and D⋯A distances (*i.e*. 3.2–2.2 Å and 4.0–3.2 Å, respectively) and of the D–H–A angle (*i.e*. 90–150°) that are typical of the weak hydrogen bonds [for more details, see the hydrogen-bond classification described by Gilli & Gilli (2009 ▸)]. Our findings are similar to what has been observed for kaersutite and trioctahedral layer silicates, in which the donor is an oxygen site shared by three adjacent octahedra (belonging to the octahedral sheet) and the acceptors are oxygens of the superimposed six-membered ring of tetrahedra (belonging to the tetrahedral sheet) (Gatta *et al.*, 2017 ▸).

## 4. Discussion   

To the best of our knowledge, this is the first time that crystal structure refinement of an asbestos fibre found in the human lung environment has been accomplished. Specifically, we have obtained the structure model of a 30 µm long amosite fibre that has remained in a human lung for ∼40 years. Two sets of data were collected focusing the X-ray beam at the top edge and at the centre of the fibre, with the aim of investigating the occurrence of structural changes in the fibre.

The atomic distribution along the fibre did not change during 40 years *in vivo*. The two refinements, in the area at the top edge and at centre of the fibre, show nearly identical chemical structures with the following calculated chemical formulas: Ca_0.18_Fe_5.40_Mg_1.25_Al_0.17_Si_8_O_22_(OH_1.92_F_0.08_) (top) and Ca_0.17_Fe_5.30_Mg_1.31_Al_0.22_Si_8_O_22_(OH)_2_ (centre).

The refined structure models show that the two independent tetrahedra do not contain Al as the values of the mean tetrahedral distances, 〈*T*(1)–O〉 = 1.616 (4) Å and 〈*T*(2)–O〉 = 1.624 (4) Å, are consistent with the literature values for virtually Al-free *C*2/*m* amphiboles (Hawthorne & Oberti, 2007 ▸). In agreement with the literature data (Finger, 1969 ▸; Pollastri *et al.*, 2015 ▸), Fe atoms populate the octahedral and distorted octahedral *M*(1), *M*(2), *M*(3) and *M*(4) sites in the structure. Mg atoms substitute for Fe in the *M*(1) and *M*(2) sites, whereas Al and Ca substitute for Fe in the *M*(3) and *M*(4) cavities, respectively. The preference of Mg for site *M*(2) (see Table 2 ▸) is also in agreement with the literature data for cummingtonite–grunerite single crystals (Finger, 1969 ▸; Ghose & Ganguly, 1982 ▸). The Fe/(Fe + Mg) ratio calculated from the structure refinement is 0.80–0.81. Using the semi-quantitative Hirschmann *et al.* (1994 ▸) plots of the dependence of the unit-cell parameters *a, b, c* and β along the cummingtonite–grunerite series versus the Fe/(Fe + Mg) ratio, the calculated ratio is slightly lower and in the range 0.70–0.75. This difference is seemingly caused by an overestimation of iron from the structure refinement. In fact, since it was not possible to determine the actual Fe^3+^ content with Mössbauer or other spectroscopic methods, all iron was assumed to be in Fe^2+^ form and hence was intrinsically overestimated.

**Figure 6:**
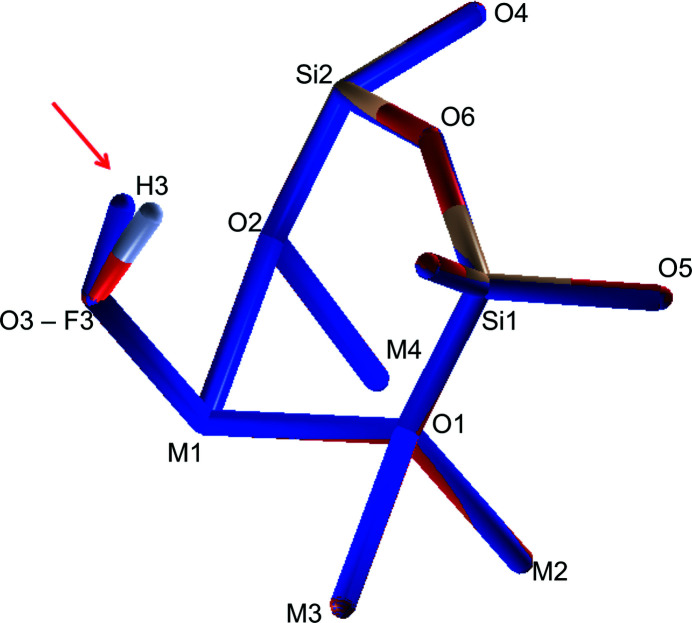
A view of the structure overlay of the refined structure of fibre_T (in colour) and fibre_C (monochromatic blue). The two structures strongly overlap on all the atomic positions; the largest distance between corresponding atoms (*i.e*. 0.296 Å) involves the hydrogen atoms (pointed out by the red arrow). In the case of fibre_T, the *W* site is characterized by an F substitution, and the atomic species partition for this site is 0.96 (3) for O and 0.04 (3) for F. The plots were designed using the *SIR2019* software (Burla *et al.*, 2015 ▸).

A small fraction [0.04 (3)%] of F atoms replaces the hydroxyl anions in the crystal lattice for the top area of the fibre. Nevertheless, as shown above, the refined crystal structure determined by the analysis of both fibre_T and fibre_C is strongly overlapping (the largest distance between corresponding atoms in the two models involves hydrogen atoms, pointed out by the red arrow in Fig. 6 ▸, which shows the overlay of the two structures) and characterized by similar site partitions of the sharing-site atoms, the F content being the only exception.

The fluorine zonation inside amphibole fibres is not surprising. It is well known from the literature that natural amphiboles may display variable contents of fluorine atoms from the centre to the edge of the crystals. This fluorine zonation in amphiboles is used as marker to gain information about the amphibole-rich rock formation in metasomatic conditions (Brabander *et al.*, 1995 ▸). These authors found that fluorine metasomatism by volume diffusion in amphiboles is relatively slow and the Arrhenius relationships for F ↔ OH inter-diffusion allow one to extract information about the maximum duration of metasomatic or thermal events from fluorine-zoned amphiboles. The slight variation observed from the centre to the edge of our amosite crystal fibre probably reflects the original zonation of the crystal at the time of formation.

The results of our study deliver undisputable evidence that amphibole asbestos fibres like amosite and crocidolite can be chemically stable at the atomic scale in the lungs for ∼40 years, whereas chrysotile asbestos progressively dissolves into a silica-rich product that is amorphous to diffraction. The first implication of our results is that amphibole asbestos fibres release small to null amounts of iron or other toxic ions when they are internalized within the cells. Hence, a direct toxic action explained by the so-called ‘Trojan horse model’ (Sabella *et al.*, 2014 ▸), observed for some families of nanoparticles, is ruled out.

The second relevant implication of the observed structural resilience is that, according to the ‘chronic inflammation theory’, the fibres do prompt persistent activation of phagocytic cells (namely macrophages), and their frustrated phagocytosis induces chronic inflammation *via* the release of highly reactive species such as reactive oxygen and nitro­gen species. These biochemical processes provoke damage of the tissues and the initiation of adverse effects leading to carcinogenesis (Yang *et al.*, 2006 ▸; Toyokuni, 2009 ▸; Donaldson *et al.*, 2010 ▸). Therefore, the classification of amphibole asbestos as a potent toxic agent and carcinogen for humans is fully justified, delivering a robust scientific base for its universal ban.

The third implication of our work is that our structure refinements clearly show that the amosite fibres are not iron-depleted. Atomic diffusion does not take place along the length of the fibre and the source of the elements forming the ABs is not from within the fibre. The formation process of ABs involves the coating of fibres with a thick layer of iron–protein–mucopolysaccharide material (Roggli, 2014 ▸; Capella *et al.*, 2017 ▸). Although some studies suggested that these ABs are formed by diffusion of dissolved substances from the fibre surface (see, for example, Suzuki & Churg, 1969 ▸), it is deemed that the fibre-coating process represents the exocytotic activity of AMs aimed at isolating the toxic reactive fibres by deposition of ferroproteins (*e.g.* ferritin and hemosiderin) onto the fibre surface (Di Giuseppe *et al.*, 2019 ▸). Prompted by the results of an in-depth study of ABs found in the lung tissue of a patient diagnosed with MM, Di Giuseppe *et al.* (2019 ▸) proposed a model for the formation mechanism of ABs. Asbestos fibres reaching the alveolar space promote the recruitment of AMs. Although the phagocytosis mechanism is effective, it fails for long fibres (Stanton *et al.*, 1981 ▸). Lung cells subject to prolonged exposure to asbestos fibres induce the release of cytoprotective agents such as ferritin (Arosio & Levi, 2002 ▸; Capella *et al.*, 2017 ▸). At this stage, when the AMs undergo frustrated phagocytosis and inflammatory activity, the formation of ABs, the last body-defence mechanism, takes place and ferritin is deposited onto the fibres by the AMs. The results of our study endorse this model, demonstrating that the iron pool for the formation of the ABs cannot be the asbestos fibres themselves but instead is biological (haemoglobin/plasma derived).

## 5. Conclusions   

In this work, we have assessed for the first time the *in vivo* stability of asbestos fibres at the atomic level. The stability of the atomic structure of an amosite amphibole asbestos fibre that had remained for ∼40 years in the lungs of a subject originally exposed to a mixture of chrysotile, amosite and crocidolite has been proven. Regarding chrysotile fibres, we only found relicts of the original structures that were amorphous and magnesium depleted. Amphibole fibres that were recovered and suitable for synchrotron X-ray micro-diffraction experiments were undamaged. The crystal structure refinement from a recovered amosite fibre shows that the original atomic distribution in the crystal is intact. These results have paramount importance for the understanding of the asbestos toxicity/carcinogenicity mechanisms as they show that the atomic structure of amphibole asbestos fibres remains stable in the lungs for a lifetime, during which they can cause chronic inflammation and other adverse effects that are responsible for carcinogenesis.

This state-of-the-art study was possible by using synchrotron micro-diffraction. The ESRF has just been upgraded to ‘Extremely Bright Source’ (EBS) (Dimper *et al.*, 2014 ▸) from a third- to a fourth-generation synchrotron radiation source with an increase in brightness by more than an order of magnitude for the materials science beamline ID11. For the first results of the ID11 beamline commissioning, the ultimate dimension of 90 nm in the vertical focusing was reached. The possibility of tuning the photon energy (between 35 and 75 keV) and the reduction in the size of the X-ray sources, making the beam even less divergent, will enable further future systematic study for unambiguous structure solutions of fibres directly in the organic media. By performing similar structural analysis without extracting the crystalline fibres from the tissues, we expect to close the gap between the structural and chemical properties of the asbestos fibres and their toxicity.

## Supplementary Material

Crystal structure: contains datablock(s) I, II, shelx. DOI: 10.1107/S2052252520015079/ro5022sup1.cif

Supporting information. DOI: 10.1107/S2052252520015079/ro5022sup2.pdf

CCDC references: 2046721, 2046722, 2046723

## Figures and Tables

**Table 1:** Crystal data, data collection and structure refinement for fibre_T and fibre_C Computer programs: *SHELXL2014/7* (Sheldrick, 2015 ▸).Geometry. All ESDs [except the ESD in the dihedral angle between two least-squares (l.s.) planes] are estimated using the full covariance matrix. The cell ESDs are taken into account individually in the estimation of ESDs in distances, angles and torsion angles; correlations between ESDs in cell parameters are only used when they are defined by crystal symmetry. An approximate (isotropic) treatment of cell ESDs is used for estimating ESDs involving l.s. planes.Refinement. Refinement of *F* ^2^ against all reflections. The weighted *R*-factor *wR* and goodness of fit *S* are based on *F* ^2^, conventional *R*-factors *R* are based on *F*, with *F* set to zero for negative *F* ^2^. The threshold expression of *F* ^2^ > 2σ(*F* ^2^) is only used for calculating *R* factors, *e.g.* *R*[*F* ^2^ > 2σ(*F* ^2^)], and is not relevant to the choice of reflections for refinement. *R* factors based on *F* ^2^ are statistically about twice as large as those based on *F*, and *R* factors based on all data will be even larger.

	fibre_T	fibre_C
Crystal data		
Chemical formula	Ca_0.18_Fe_5.40_Mg_1.25_Al_0.17_Si_8_O_23.92_F_0.08_H_1.92_	Ca_0.17_Fe_5.30_Mg_1.31_Al_0.22_Si_8_O_24_H_2_
*M*_r_	954.66	951.39
Crystal system, space group	Monoclinic, *C*2/*m*	Monoclinic, *C*2/*m*
Temperature (K)	293	293
*a, b, c* (Å)	9.5440 (2), 18.2455 (4), 5.32660 (12)	9.5343 (2), 18.2210 (4), 5.3202 (2)
β (°)	101.883 (2)	101.846 (3)
*V* (Å^3^)	907.67 (3)	904.57 (4)
*Z*	2	2
Radiation type, wavelength (Å)	Synchrotron, λ = 0.30909	Synchrotron, λ = 0.30911
Photon flux (s^−1^)	1.88 × 10^6^	1.88 × 10^6^
μ (mm^−1^)	0.5	0.5
Crystal size (mm)	0.001 × 0.001 × 0.001	0.001 × 0.001 × 0.001
		
Data collection		
Diffractometer	ID11 nanoscope	ID11 nanoscope
Absorption correction	Multi-scan *SCALE3 ABSPACK*; Oxford Diffraction (2006 ▸)	Multi-scan *SCALE3 ABSPACK*; Oxford Diffraction (2006 ▸)
No. of measured, independent and observed [*I* > 2σ(*I*)] reflections	7498, 1089, 986	6629, 1090, 1044
*R*_int_	0.030	0.047
(sin θ/λ)_max_ (Å^−1^)	0.666	0.666
		
Refinement		
*R*[*F* ^2^ > 2σ(*F* ^2^)], *wR*(*F* ^2^), *S*	0.045, 0.159, 1.30	0.054, 0.135, 1.23
No. of reflections	1089	1090
No. of parameters	107	105
No. of restraints	5	4
Hydrogen atom treatment	Only hydrogen atom coordinates refined *w* = 1/[σ^2^(*F*_o_^2^) + (0.0581*P*)^2^ + 17.3651*P*] where *P* = (*F*_o_^2^ + 2*F*_c_^2^)/3	Only hydrogen atom coordinates refined *w* = 1/[σ^2^(*F*_o_^2^) + (0.0301*P*)^2^ + 27.1117*P*] where *P* = (*F*_o_^2^ + 2*F*_c_^2^)/3
Δρ_max_, Δρ_min_ (e Å^−3^)	1.27, −0.95	1.12, −1.20

**Table 2:** Final atomic coordinates, occupancies, and atomic displacement parameters (Å^2^) for fibre_T and fibre_C

	fibre_T					fibre_C				
Atom	*x*/*a*	*y*/*b*	*z*/*c*	*B*	Site partition	*x*/*a*	*y*/*b*	*z*/*c*	*B*	Site partition
*M*(1)	0.0000	0.08535 (7)	0.5000	0.0120 (5)	0.763 (13) Fe^2+^	0.0000	0.08522 (7)	0.5000	0.0105 (5)	0.744 (13) Fe^2+^
					0.237 (13) Mg					0.256 (13) Mg
*M*(2)	0.0000	0.17889 (8)	0.0000	0.0105 (5)	0.615 (12) Fe^2+^	0.0000	0.17901 (8)	0.0000	0.0091 (5)	0.600 (13) Fe^2+^
					0.385 (13) Mg					0.400 (13) Mg
*M*(3)	0.0000	0.0000	0.0000	0.0135 (6)	0.827 (18) Fe^3+^	0.0000	0.0000	0.0000	0.0117 (6)	0.785 (19) Fe^3+^
					0.173 (19) Al^3+^					0.215 (19) Al^3+^
*M*(4)	0.0000	0.25861 (7)	0.5000	0.0141 (4)	0.908 (19) Fe^2+^	0.0000	0.25857 (7)	0.5000	0.0128 (4)	0.914 (19) Fe^2+^
					0.092 (19) Ca					0.086 (19) Ca^2+^
*T*(1)	0.28717 (16)	0.08369 (8)	0.2703 (3)	0.0091 (4)		0.28718 (17)	0.08369 (8)	0.2701 (3)	0.0081 (4)	
*T*(2)	0.29771 (16)	0.16760 (8)	0.7762 (3)	0.0095 (4)		0.29777 (17)	0.16759 (8)	0.7760 (3)	0.0085 (4)	
O(1)	0.1144 (4)	0.0878 (2)	0.2077 (7)	0.0118 (8)		0.1149 (4)	0.0878 (2)	0.2083 (8)	0.0112 (8)	
O(2)	0.1247 (4)	0.1727 (2)	0.7136 (7)	0.0124 (8)		0.1251 (4)	0.1725 (2)	0.7137 (8)	0.0121 (8)	
O(3)–OH	0.1100 (7)	0.0000	0.7073 (11)	0.0165 (12)	0.96 (3) O	0.1098 (8)	0.0000	0.7072 (12)	0.0172 (13)	1 O
					0.04 (3) F					
H3	0.213 (13)	0.0000	0.72 (2)	0.020	0.96 (3)	0.203 (14)	0.0000	0.77 (2)	0.021	
O(4)	0.3814 (4)	0.2436 (2)	0.7696 (7)	0.0143 (8)		0.3815 (5)	0.2436 (2)	0.7696 (8)	0.0130 (8)	
O(5)	0.3500 (4)	0.1286 (2)	0.0553 (7)	0.0134 (8)		0.3499 (4)	0.1285 (2)	0.0553 (8)	0.0119 (8)	
O(6)	0.3504 (4)	0.1202 (2)	0.5494 (7)	0.0150 (8)		0.3503 (5)	0.1204 (2)	0.5488 (8)	0.0140 (9)	
O(7)	0.3411 (6)	0.0000	0.2746 (11)	0.0155 (11)		0.3405 (6)	0.0000	0.2742 (12)	0.0142 (12)	

**Table 3:** Selected bond distances (in Å) and polyhedral distortion (Δ × 10^−4^) for fibre_T and fibre_C Note: polyhedron distortion Δ as defined by Shannon (1976 ▸), Δ = (1/*n*)Σ[(*R*_i_−*R*)/*R*]^2^, where *n* is the number of ligands, *R* is the average bond length and *R*_i_ is an individual bond length.

		fibre_T	fibre_C			fibre_T	fibre_C
*T*(1)	O(1)	1.616 (4)	1.610 (4)	*T*(2)	O(2)	1.618 (4)	1.614 (4)
	O(5)	1.620 (4)0	1.616 (4)		O(4)	1.604 (4)	1.603 (4)
	O(6)	1.627 (4)	1.625 (4)		O(5)	1.631 (4)	1.631 (4)
	O(7)	1.610 (2)	1.606 (2)		O(6)	1.647 (4)	1.643 (4)
	Average	1.618 (4)	1.614 (4)		Average	1.625 (4)	1.623 (4)
	Δ	0.146	0.196		Δ	0.955	0.899
*M*(1)	O(1) × 2	2.078 (4)	2.076 (4)	*M*(2)	O(1) × 2	2.163 (4)	2.165 (4)
	O(2) × 2	2.167 (4)	2.165 (4)		O(2) × 2	2.122 (4)	2.122 (4)
	O(3) × 2	2.066 (4)	2.061 (5)		O(4) × 2	2.053 (4)	2.049 (4)
	Average	2.104 (4)	2.101 (4)		Average	2.113 (4)	2.112 (4)
	Δ	4.670	4.652		Δ	4.688	5.223
*M*(3)	O(1) × 4	2.118 (4)	2.119 (4)	*M*(4)	O(2) × 2	2.147 (4)	2.149 (4)
	O(3) × 2	2.051 (6)	2.047 (6)		O(4) × 2	2.004 (4)	2.000 (4)
					O(6) × 2	2.674 (4)	2.667 (5)
	Average	2.096 (5)	2.095 (5)		Average	2.275 (4)	2.272 (4)
	Δ	2.324	2.688		Δ	147.021	145.692
