# Supplementary material for: Crystal structure determination of a lifelong biopersistent asbestos fibre using single-crystal synchrotron X-ray micro-diffraction
Source: IUCrJ. 2021 Jan 1;8(Pt 1):76–86. doi: 10.1107/S2052252520015079 (PMC7792997; doi:10.1107/S2052252520015079)
Supplement: Supplementary file 2 [file m-08-00076-sup2.pdf]

IUCrJ

Volume 8 (2021)

Supporting information for article:

Crystal structure determination of a lifelong biopersistent asbestos fibre using single-crystal synchrotron X-ray micro-diffraction

Carlotta Giacobbe, Dario Di Giuseppe, Alessandro Zoboli, Magdalena Lassinantti Gualtieri, Paola Bonasoni, Anna Moliterni, Nicola Corriero, Angela Altomare, Jonathan Wright and Alessandro F. Gualtieri

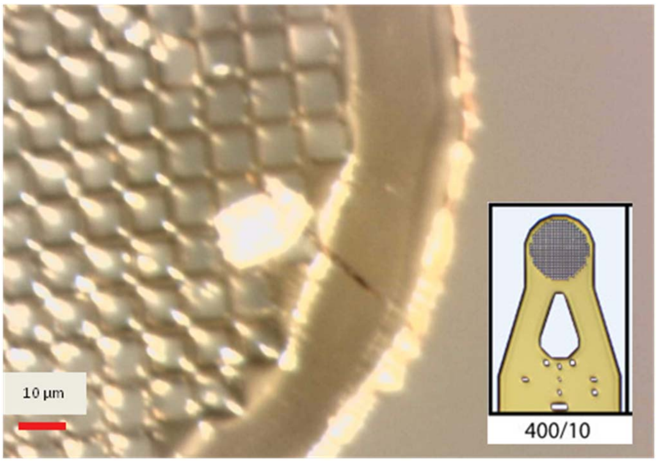

Figure S1 The amosite fibre mounted on a MiTeGen microloops^{TM} of 400 μm diameter and 10 μm mesh size.

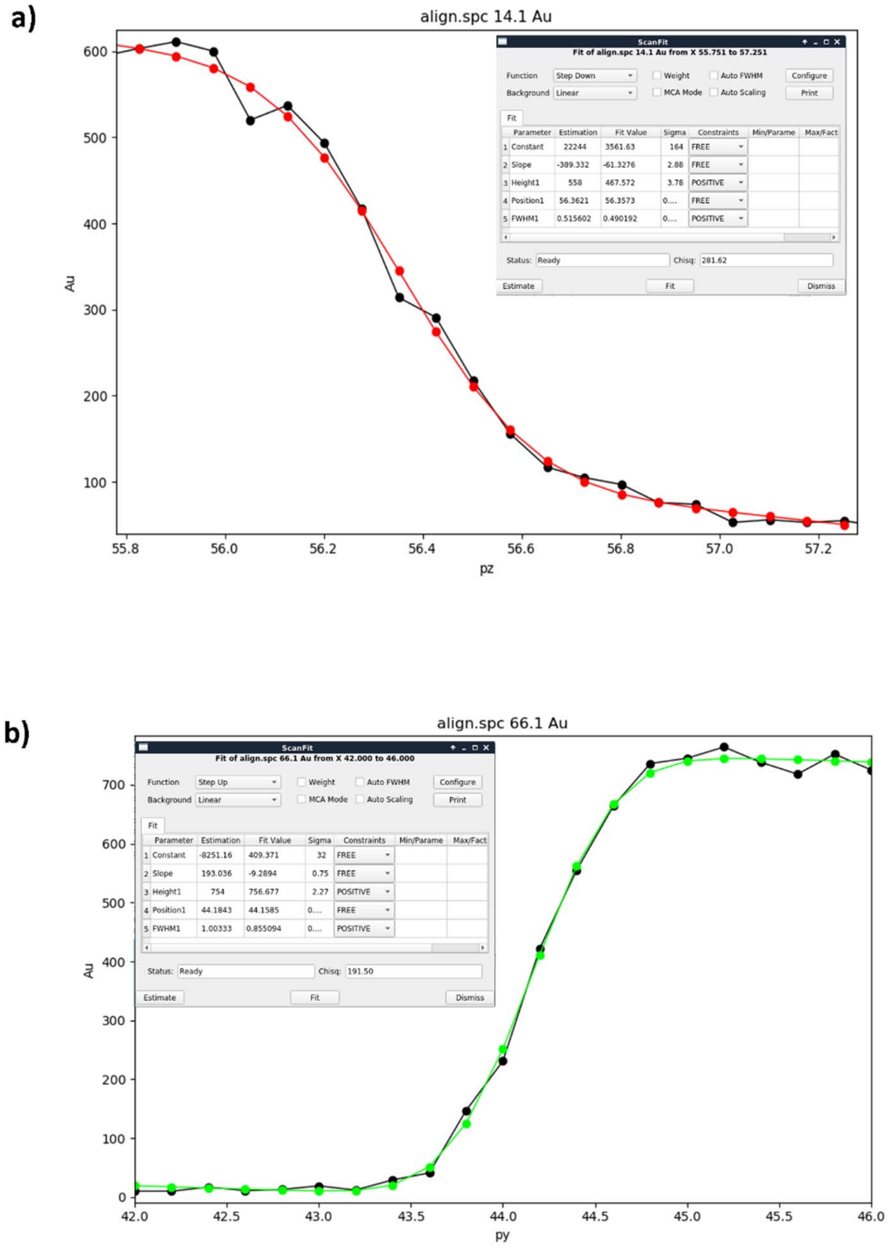

Figure S2 Fit of the “knife edge scans” of a Tungsten wire, plated with 3–5% by weight gold coating across the beam. Fit (a) of the wire in the vertical dimension allowed an estimation of the beam size of 490 nm. Fit (b) of the wire in the horizontal dimension allowed an estimation of the beam size of 850 nm.

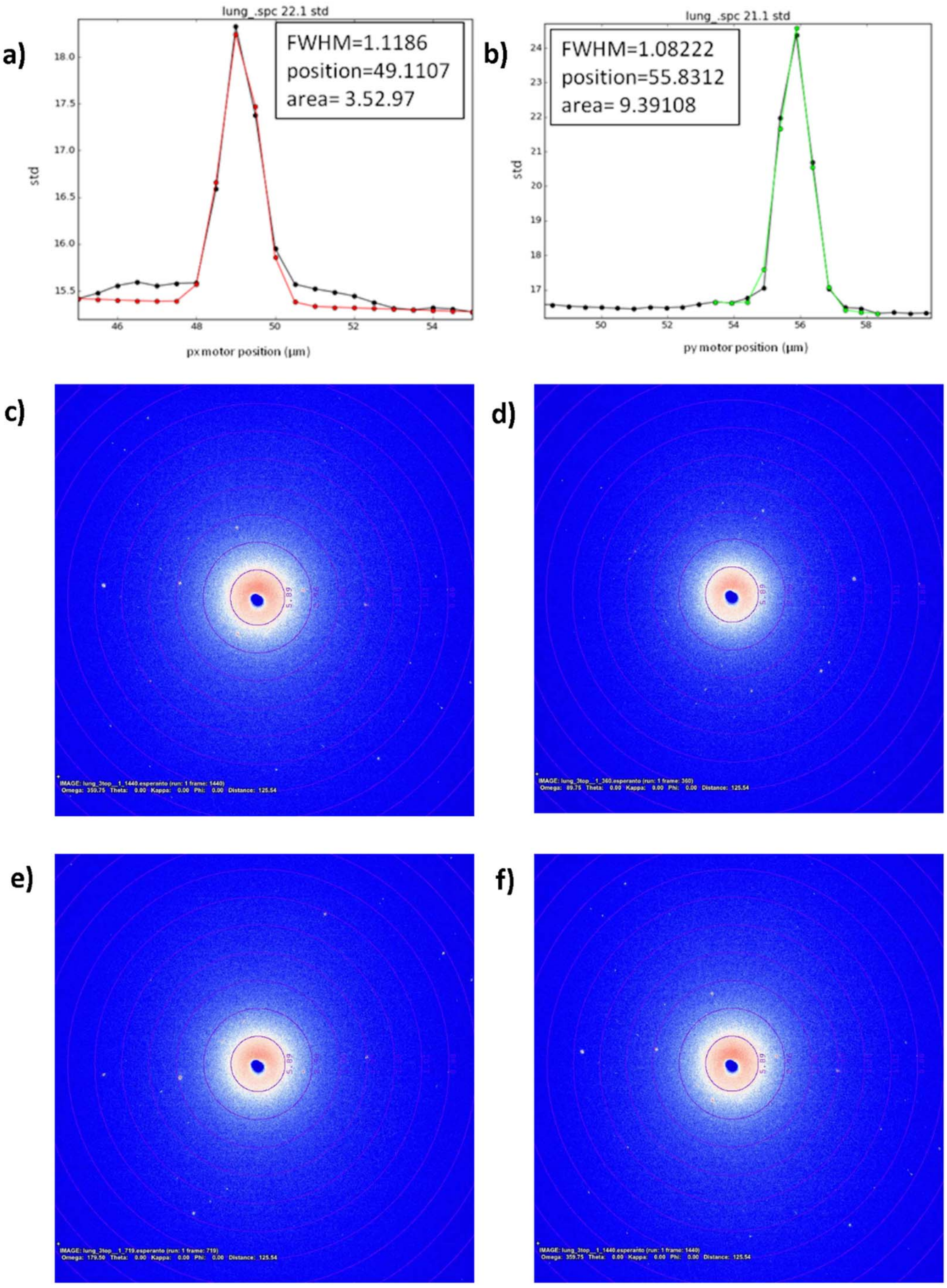

Figure S3 (a) Fit of the crystal size of the amphibole fibre by scanning the motor "px" across the beam (x dimension of the fibre). (b) Fit of the crystal size of the amphibole fibre by scanning the motor "py" across the beam, after a rotation at 90 degrees on z (y dimension of the fibre). (c) Single crystal diffraction image of the amphibole fibre at rotation ω= 0°. (d) Single crystal diffraction image of the amphibole fibre at rotation ω = 90°. (e) Single crystal diffraction image of the amphibole fibre at rotation ω = 180°. (f) Single crystal diffraction image of the amphibole fibre at rotation ω= 270°. In each of these images diffraction spots are present indicating the crystalline nature of the fibre.

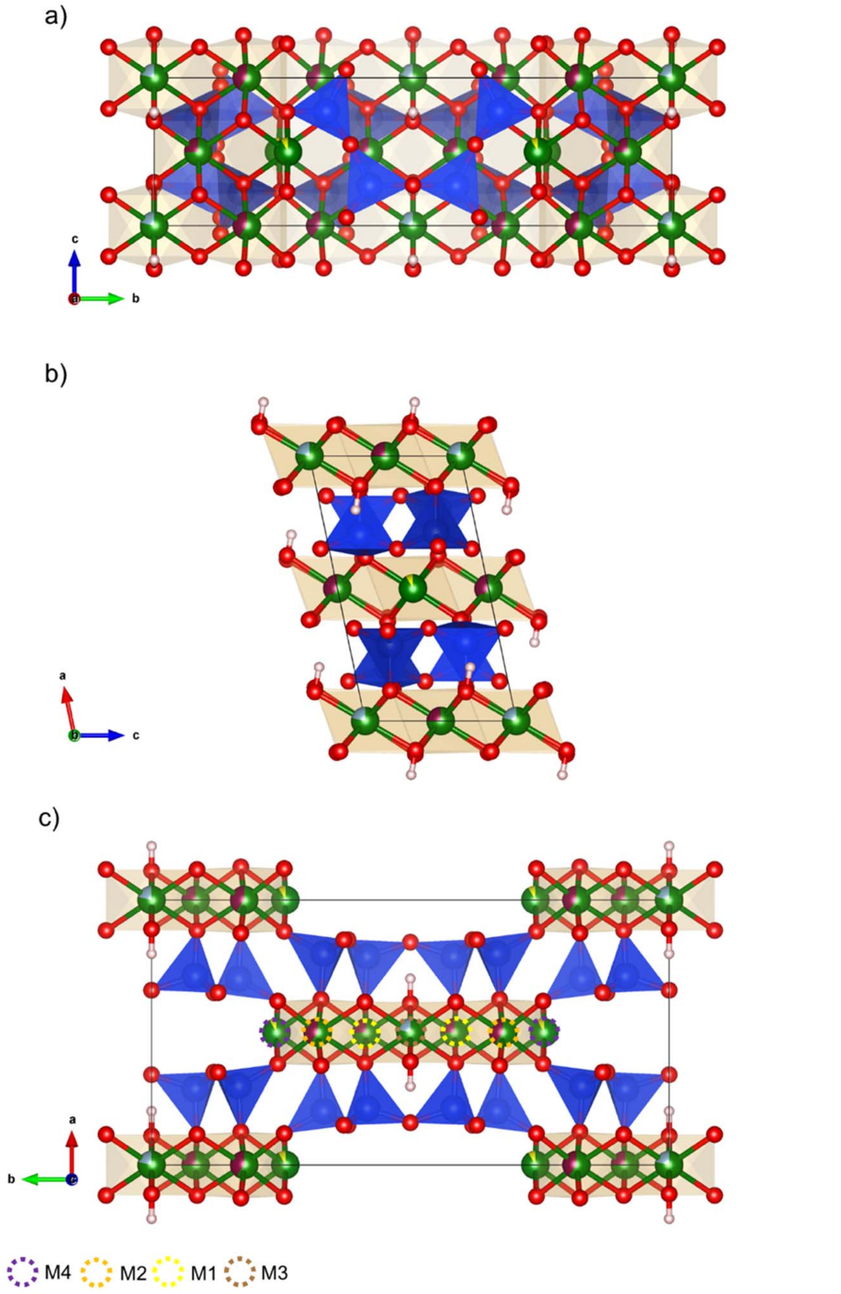

Figure S4 Structure model of fibrous amosite (fibre_C) . Projection along *a* (a). Projection along *b* (b). Projection along *c* (c). Legend: blue polyhedra = tetrahedral centred by Si; blue balls= Si atoms; green balls= Fe ; red balls = O atoms; purple balls= Mg atoms; yellow balls = Ca atoms, light blue balls= F substituting O atoms. The dotted line circles are used to better illustrate the amphibole sites (Hawthorne, 1983). The plots were created using the VESTA software (Momma & Izumi, 2011).

Table S1 Selected geometric parameters (Å, º) for fibre_T.

Fe1—O3	2.066 (4)	Fe4—O4^{iv}	2.004 (4)
Fe1—F3^{i}	2.066 (4)	Fe4—O2^{ii}	2.147 (4)
Fe1—O3^{i}	2.066 (4)	Fe4—O2	2.147 (4)
Fe1—O1	2.078 (4)	Fe4—Mg2^{iii}	3.0346 (9)
Fe1—O1^{ii}	2.078 (4)	Si1—O7	1.610 (2)
Fe1—O2^{ii}	2.167 (4)	Si1—O1	1.616 (4)
Fe1—O2	2.167 (4)	Si1—O5	1.620 (4)
Fe1—Mg1^{i}	3.114 (3)	Si1—O6	1.627 (4)
Fe1—Mg2^{iii}	3.1632 (10)	Si1—Ca4^{iv}	3.5888 (17)
Fe2—O4^{iv}	2.053 (4)	Si2—O4	1.604 (4)
Fe2—O4^{v}	2.053 (4)	Si2—O2	1.618 (4)
Fe2—O2^{vi}	2.122 (4)	Si2—O5^{iii}	1.631 (4)
Fe2—O2^{ii}	2.122 (4)	Si2—O6	1.647 (4)
Fe2—O1	2.163 (4)	Si2—Ca4^{iv}	2.9791 (15)
Fe2—O1^{vii}	2.163 (4)	O2—Mg2^{iii}	2.123 (4)
Fe2—Ca4^{vi}	3.0346 (9)	O2—Fe2^{iii}	2.123 (4)
Fe2—Mg1^{vi}	3.1632 (10)	O3—Al3^{iii}	2.051 (6)
Fe3—F3^{i}	2.051 (6)	O3—Fe3^{iii}	2.051 (6)
Fe3—F3^{vi}	2.051 (6)	O3—Fe1^{i}	2.066 (4)
Fe3—O3^{i}	2.051 (6)	O3—Mg1^{i}	2.066 (4)
Fe3—O3^{vi}	2.051 (6)	O3—H3	0.97 (12)
Fe3—O1^{viii}	2.118 (4)	O4—Ca4^{iv}	2.004 (4)
Fe3—O1^{ix}	2.118 (4)	O4—Fe4^{iv}	2.004 (4)
Fe3—O1^{vii}	2.118 (4)	O4—Fe2^{iv}	2.053 (4)
Fe3—O1	2.118 (4)	O4—Mg2^{iv}	2.053 (4)
Fe3—Mg1^{viii}	3.0852 (6)	O5—Si2^{vi}	1.631 (4)
Fe3—Mg1^{i}	3.0852 (6)	O6—Ca4^{iv}	2.674 (4)
Fe3—Mg1^{vi}	3.0852 (6)	O7—Si1^{ix}	1.610 (2)
Fe4—O4^{x}	2.004 (4)		
O3—Fe1—F3^{i}	82.2	O1^{vii}—Fe3—Mg1^{viii}	91.28 (10)

O3—Fe1—O3^{i}	82.2 (3)	O1—Fe3—Mg1^{viii}	137.84 (10)
F3^{i}—Fe1—O3^{i}	0.0	F3^{i}—Fe3—Mg1^{i}	41.66 (11)
O3—Fe1—O1	97.42 (19)	F3^{vi}—Fe3—Mg1^{i}	138.34 (11)
F3^{i}—Fe1—O1	84.45 (19)	O3^{i}—Fe3—Mg1^{i}	41.66 (11)
O3^{i}—Fe1—O1	84.45 (19)	O3^{vi}—Fe3—Mg1^{i}	138.34 (11)
O3—Fe1—O1^{ii}	84.45 (19)	O1^{viii}—Fe3—Mg1^{i}	88.72 (10)
F3^{i}—Fe1—O1^{ii}	97.42 (19)	O1^{ix}—Fe3—Mg1^{i}	42.16 (10)
O3^{i}—Fe1—O1^{ii}	97.42 (19)	O1^{vii}—Fe3—Mg1^{i}	137.84 (10)
O1—Fe1—O1^{ii}	177.5 (2)	O1—Fe3—Mg1^{i}	91.28 (10)
O3—Fe1—O2^{ii}	177.28 (18)	Mg1^{viii}—Fe3—Mg1^{i}	119.37 (4)
F3^{i}—Fe1—O2^{ii}	96.32 (17)	F3^{i}—Fe3—Mg1^{vi}	138.34 (11)
O3^{i}—Fe1—O2^{ii}	96.32 (17)	F3^{vi}—Fe3—Mg1^{vi}	41.66 (11)
O1—Fe1—O2^{ii}	84.68 (14)	O3^{i}—Fe3—Mg1^{vi}	138.34 (11)
O1^{ii}—Fe1—O2^{ii}	93.50 (15)	O3^{vi}—Fe3—Mg1^{vi}	41.66 (11)
O3—Fe1—O2	96.32 (17)	O1^{viii}—Fe3—Mg1^{vi}	91.28 (10)
F3^{i}—Fe1—O2	177.28 (18)	O1^{ix}—Fe3—Mg1^{vi}	137.84 (10)
O3^{i}—Fe1—O2	177.28 (18)	O1^{vii}—Fe3—Mg1^{vi}	42.16 (10)
O1—Fe1—O2	93.50 (15)	O1—Fe3—Mg1^{vi}	88.72 (10)
O1^{ii}—Fe1—O2	84.68 (15)	Mg1^{viii}—Fe3—Mg1^{vi}	60.63 (4)
O2^{ii}—Fe1—O2	85.3 (2)	Mg1^{i}—Fe3—Mg1^{vi}	180.00 (4)
O3—Fe1—Mg1^{i}	41.10 (13)	O4^{x}—Fe4—O4^{iv}	177.7 (2)
F3^{i}—Fe1—Mg1^{i}	41.10 (13)	O4^{x}—Fe4—O2^{ii}	91.83 (16)
O3^{i}—Fe1—Mg1^{i}	41.10 (13)	O4^{iv}—Fe4—O2^{ii}	86.50 (15)
O1—Fe1—Mg1^{i}	91.24 (11)	O4^{x}—Fe4—O2	86.51 (15)
O1^{ii}—Fe1—Mg1^{i}	91.23 (11)	O4^{iv}—Fe4—O2	91.82 (15)
O2^{ii}—Fe1—Mg1^{i}	137.37 (11)	O2^{ii}—Fe4—O2	86.2 (2)
O2—Fe1—Mg1^{i}	137.37 (11)	O4^{x}—Fe4—Mg2^{iii}	42.21 (11)
O3—Fe1—Mg2^{iii}	92.56 (13)	O4^{iv}—Fe4—Mg2^{iii}	136.19 (12)
F3^{i}—Fe1—Mg2^{iii}	140.22 (16)	O2^{ii}—Fe4—Mg2^{iii}	90.86 (11)
O3^{i}—Fe1—Mg2^{iii}	140.22 (16)	O2—Fe4—Mg2^{iii}	44.38 (10)
O1—Fe1—Mg2^{iii}	135.26 (11)	O7—Si1—O1	110.9 (2)

$O1^{ii}$—Fe1—$Mg2^{iii}$	42.81 (11)	O7—Si1—O5	109.0 (3)
$O2^{ii}$—Fe1—$Mg2^{iii}$	87.12 (11)	O1—Si1—O5	110.0 (2)
O2—Fe1—$Mg2^{iii}$	41.95 (10)	O7—Si1—O6	108.5 (3)
$Mg1^{i}$—Fe1—$Mg2^{iii}$	122.65 (3)	O1—Si1—O6	109.9 (2)
$O4^{iv}$—Fe2—$O4^{v}$	92.9 (2)	O5—Si1—O6	108.5 (2)
$O4^{iv}$—Fe2—$O2^{vi}$	98.30 (16)	O7—Si1—$Ca4^{iv}$	126.8 (2)
$O4^{v}$—Fe2—$O2^{vi}$	85.90 (15)	O1—Si1—$Ca4^{iv}$	121.01 (15)
$O4^{iv}$—Fe2—$O2^{ii}$	85.90 (15)	O5—Si1—$Ca4^{iv}$	64.53 (15)
$O4^{v}$—Fe2—$O2^{ii}$	98.30 (16)	O6—Si1—$Ca4^{iv}$	44.19 (15)
$O2^{vi}$—Fe2—$O2^{ii}$	173.9 (2)	O4—Si2—O2	115.9 (2)
$O4^{iv}$—Fe2—O1	93.76 (15)	O4—Si2—$O5^{iii}$	109.7 (2)
$O4^{v}$—Fe2—O1	173.16 (16)	O2—Si2—$O5^{iii}$	108.7 (2)
$O2^{vi}$—Fe2—O1	91.63 (15)	O4—Si2—O6	102.1 (2)
$O2^{ii}$—Fe2—O1	83.70 (15)	O2—Si2—O6	109.4 (2)
$O4^{iv}$—Fe2—$O1^{vii}$	173.16 (16)	$O5^{iii}$—Si2—O6	110.9 (2)
$O4^{v}$—Fe2—$O1^{vii}$	93.76 (15)	O4—Si2—$Ca4^{iv}$	38.94 (15)
$O2^{vi}$—Fe2—$O1^{vii}$	83.70 (15)	O2—Si2—$Ca4^{iv}$	127.54 (15)
$O2^{ii}$—Fe2—$O1^{vii}$	91.63 (15)	$O5^{iii}$—Si2—$Ca4^{iv}$	122.68 (15)
O1—Fe2—$O1^{vii}$	79.6 (2)	O6—Si2—$Ca4^{iv}$	63.13 (15)
$O4^{iv}$—Fe2—$Ca4^{vi}$	95.43 (11)	Si1—O1—Fe1	121.1 (2)
$O4^{v}$—Fe2—$Ca4^{vi}$	40.97 (11)	Si1—O1—Fe3	117.8 (2)
$O2^{vi}$—Fe2—$Ca4^{vi}$	45.02 (11)	Fe1—O1—Fe3	94.67 (16)
$O2^{ii}$—Fe2—$Ca4^{vi}$	139.25 (11)	Si1—O1—Fe2	121.8 (2)
O1—Fe2—$Ca4^{vi}$	136.55 (10)	Fe1—O1—Fe2	96.45 (16)
$O1^{vii}$—Fe2—$Ca4^{vi}$	90.60 (10)	Fe3—O1—Fe2	99.35 (16)
$O4^{iv}$—Fe2—$Mg1^{vi}$	141.20 (11)	Si2—O2—$Mg2^{iii}$	123.6 (2)
$O4^{v}$—Fe2—$Mg1^{vi}$	87.94 (11)	Si2—O2—$Fe2^{iii}$	123.6 (2)
$O2^{vi}$—Fe2—$Mg1^{vi}$	43.03 (11)	$Mg2^{iii}$—O2—$Fe2^{iii}$	0.0
$O2^{ii}$—Fe2—$Mg1^{vi}$	132.37 (11)	Si2—O2—Fe4	125.6 (2)
O1—Fe2—$Mg1^{vi}$	85.91 (11)	$Mg2^{iii}$—O2—Fe4	90.60 (15)
$O1^{vii}$—Fe2—$Mg1^{vi}$	40.74 (10)	$Fe2^{iii}$—O2—Fe4	90.60 (15)

$Ca4^{vi}—Fe2—Mg1^{vi}$	61.29 (3)	Si2—O2—Fe1	119.5 (2)
$F3^{i}—Fe3—F3^{vi}$	180.0 (3)	$Mg2^{iii}—O2—Fe1$	95.02 (15)
$F3^{i}—Fe3—O3^{i}$	0.0	$Fe2^{iii}—O2—Fe1$	95.02 (15)
$F3^{vi}—Fe3—O3^{i}$	180.0	Fe4—O2—Fe1	94.25 (15)
$F3^{i}—Fe3—O3^{vi}$	180.0	$Al3^{iii}—O3—Fe3^{iii}$	0.0
$F3^{vi}—Fe3—O3^{vi}$	0.0	$Al3^{iii}—O3—Fe1$	97.1 (2)
$O3^{i}—Fe3—O3^{vi}$	180.0 (3)	$Fe3^{iii}—O3—Fe1$	97.1 (2)
$F3^{i}—Fe3—O1^{viii}$	96.18 (15)	$Al3^{iii}—O3—Fe1^{i}$	97.1 (2)
$F3^{vi}—Fe3—O1^{viii}$	83.82 (15)	$Fe3^{iii}—O3—Fe1^{i}$	97.1 (2)
$O3^{i}—Fe3—O1^{viii}$	96.18 (15)	$Fe1—O3—Fe1^{i}$	97.8 (3)
$O3^{vi}—Fe3—O1^{viii}$	83.82 (15)	$Al3^{iii}—O3—Mg1^{i}$	97.1 (2)
$F3^{i}—Fe3—O1^{ix}$	83.82 (15)	$Fe3^{iii}—O3—Mg1^{i}$	97.1 (2)
$F3^{vi}—Fe3—O1^{ix}$	96.18 (15)	$Fe1—O3—Mg1^{i}$	97.8
$O3^{i}—Fe3—O1^{ix}$	83.82 (15)	$Fe1^{i}—O3—Mg1^{i}$	0.00 (5)
$O3^{vi}—Fe3—O1^{ix}$	96.18 (15)	$Al3^{iii}—O3—H3$	129 (6)
$O1^{viii}—Fe3—O1^{ix}$	81.7 (2)	$Fe3^{iii}—O3—H3$	129 (6)
$F3^{i}—Fe3—O1^{vii}$	96.18 (15)	Fe1—O3—H3	115 (4)
$F3^{vi}—Fe3—O1^{vii}$	83.82 (15)	$Fe1^{i}—O3—H3$	115 (4)
$O3^{i}—Fe3—O1^{vii}$	96.18 (15)	$Mg1^{i}—O3—H3$	115 (4)
$O3^{vi}—Fe3—O1^{vii}$	83.82 (15)	$Si2—O4—Ca4^{iv}$	110.8 (2)
$O1^{viii}—Fe3—O1^{vii}$	98.3 (2)	$Si2—O4—Fe4^{iv}$	110.8 (2)
$O1^{ix}—Fe3—O1^{vii}$	180.0 (2)	$Ca4^{iv}—O4—Fe4^{iv}$	0.0
$F3^{i}—Fe3—O1$	83.82 (15)	$Si2—O4—Fe2^{iv}$	142.9 (2)
$F3^{vi}—Fe3—O1$	96.18 (15)	$Ca4^{iv}—O4—Fe2^{iv}$	96.83 (16)
$O3^{i}—Fe3—O1$	83.82 (15)	$Fe4^{iv}—O4—Fe2^{iv}$	96.83 (16)
$O3^{vi}—Fe3—O1$	96.18 (15)	$Si2—O4—Mg2^{iv}$	142.9 (2)
$O1^{viii}—Fe3—O1$	180.0	$Ca4^{iv}—O4—Mg2^{iv}$	96.83 (16)
$O1^{ix}—Fe3—O1$	98.3 (2)	$Fe4^{iv}—O4—Mg2^{iv}$	96.83 (16)
$O1^{vii}—Fe3—O1$	81.7 (2)	$Fe2^{iv}—O4—Mg2^{iv}$	0.00 (6)
$F3^{i}—Fe3—Mg1^{viii}$	138.34 (11)	$Si1—O5—Si2^{vi}$	141.0 (3)
$F3^{vi}—Fe3—Mg1^{viii}$	41.66 (11)	Si1—O6—Si2	140.7 (3)

O3^{i}—Fe3—Mg1^{viii}	138.34 (11)	Si1—O6—Ca4^{iv}	110.7 (2)
O3^{vi}—Fe3—Mg1^{viii}	41.66 (11)	Si2—O6—Ca4^{iv}	83.56 (16)
O1^{viii}—Fe3—Mg1^{viii}	42.16 (10)	Si1^{ix}—O7—Si1	143.1 (4)
O1^{ix}—Fe3—Mg1^{viii}	88.72 (10)		

Symmetry code(s): (i) −*x*, −*y*, −*z*+1; (ii) −*x*, *y*, −*z*+1; (iii) *x*, *y*, *z*+1; (iv) −*x*+1/2, −*y*+1/2, −*z*+1; (v) *x*−1/2, −*y*+1/2, *z*−1; (vi) *x*, *y*, *z*−1; (vii) −*x*, *y*, −*z*; (viii) −*x*, −*y*, −*z*; (ix) *x*, −*y*, *z*; (x) *x*−1/2, −*y*+1/2, *z*.

Table S2 Selected geometric parameters (Å, º) for fibre_C.

Fe1—O3^{i}	2.061 (5)	Fe4—O2^{ii}	2.149 (4)
Fe1—O3	2.061 (5)	Fe4—O2	2.149 (4)
Fe1—O1	2.076 (4)	Fe4—Mg2^{iii}	3.0295 (9)
Fe1—O1^{ii}	2.076 (4)	Si1—O7	1.606 (2)
Fe1—O2	2.165 (4)	Si1—O1	1.610 (4)
Fe1—O2^{ii}	2.165 (4)	Si1—O5	1.616 (4)
Fe1—Mg1^{i}	3.106 (3)	Si1—O6	1.625 (4)
Fe1—Mg2^{iii}	3.1617 (11)	Si1—Ca4^{iv}	3.5852 (18)
Fe2—O4^{iv}	2.049 (4)	Si2—O4	1.603 (4)
Fe2—O4^{v}	2.049 (4)	Si2—O2	1.614 (4)
Fe2—O2^{ii}	2.122 (4)	Si2—O5^{iii}	1.631 (4)
Fe2—O2^{vi}	2.122 (4)	Si2—O6	1.643 (4)
Fe2—O1^{vii}	2.165 (4)	Si2—Ca4^{iv}	2.9741 (16)
Fe2—O1	2.165 (4)	O2—Mg2^{iii}	2.122 (4)
Fe2—Ca4^{vi}	3.0295 (9)	O2—Fe2^{iii}	2.122 (4)
Fe2—Mg1^{vi}	3.1617 (11)	O3—Al3^{iii}	2.047 (6)
Fe3—O3^{i}	2.047 (6)	O3—Fe3^{iii}	2.047 (6)
Fe3—O3^{vi}	2.047 (6)	O3—Fe1^{i}	2.061 (5)
Fe3—O1	2.119 (4)	O3—Mg1^{i}	2.061 (5)
Fe3—O1^{viii}	2.119 (4)	O3—H3	0.89 (13)
Fe3—O1^{ix}	2.119 (4)	O4—Ca4^{iv}	2.000 (4)
Fe3—O1^{vii}	2.119 (4)	O4—Fe4^{iv}	2.000 (4)

Fe3—Mg1^{viii}	3.0802 (7)	O4—Fe2^{iv}	2.049 (4)
Fe3—Mg1^{vi}	3.0802 (7)	O4—Mg2^{iv}	2.049 (4)
Fe3—Mg1^{i}	3.0802 (7)	O5—Si2^{vi}	1.631 (4)
Fe3—Mg2^{viii}	3.2617 (15)	O6—Ca4^{iv}	2.667 (5)
Fe4—O4^{x}	2.000 (4)	O7—Si1^{ix}	1.606 (2)
Fe4—O4^{iv}	2.000 (4)		
O3^{i}—Fe1—O3	82.2 (3)	O1^{vii}—Fe3—Mg1^{i}	137.79 (11)
O3^{i}—Fe1—O1	84.6 (2)	Mg1^{viii}—Fe3—Mg1^{i}	119.45 (4)
O3—Fe1—O1	97.4 (2)	Mg1^{vi}—Fe3—Mg1^{i}	180.00 (4)
O3^{i}—Fe1—O1^{ii}	97.4 (2)	O3^{i}—Fe3—Mg2^{viii}	90.0
O3—Fe1—O1^{ii}	84.6 (2)	O3^{vi}—Fe3—Mg2^{viii}	90.0
O1—Fe1—O1^{ii}	177.4 (2)	O1—Fe3—Mg2^{viii}	139.05 (11)
O3^{i}—Fe1—O2	177.2 (2)	O1^{viii}—Fe3—Mg2^{viii}	40.95 (11)
O3—Fe1—O2	96.20 (19)	O1^{ix}—Fe3—Mg2^{viii}	40.95 (11)
O1—Fe1—O2	93.31 (16)	O1^{vii}—Fe3—Mg2^{viii}	139.05 (11)
O1^{ii}—Fe1—O2	84.75 (15)	Mg1^{viii}—Fe3—Mg2^{viii}	59.73 (2)
O3^{i}—Fe1—O2^{ii}	96.20 (19)	Mg1^{vi}—Fe3—Mg2^{viii}	120.27 (2)
O3—Fe1—O2^{ii}	177.2 (2)	Mg1^{i}—Fe3—Mg2^{viii}	59.73 (2)
O1—Fe1—O2^{ii}	84.76 (15)	O4^{x}—Fe4—O4^{iv}	177.7 (2)
O1^{ii}—Fe1—O2^{ii}	93.30 (16)	O4^{x}—Fe4—O2^{ii}	91.78 (16)
O2—Fe1—O2^{ii}	85.5 (2)	O4^{iv}—Fe4—O2^{ii}	86.54 (16)
O3^{i}—Fe1—Mg1^{i}	41.12 (15)	O4^{x}—Fe4—O2	86.54 (16)
O3—Fe1—Mg1^{i}	41.12 (15)	O4^{iv}—Fe4—O2	91.78 (16)
O1—Fe1—Mg1^{i}	91.32 (12)	O2^{ii}—Fe4—O2	86.3 (2)
O1^{ii}—Fe1—Mg1^{i}	91.32 (12)	O4^{x}—Fe4—Mg2^{iii}	42.17 (12)
O2—Fe1—Mg1^{i}	137.27 (11)	O4^{iv}—Fe4—Mg2^{iii}	136.21 (13)
O2^{ii}—Fe1—Mg1^{i}	137.27 (11)	O2^{ii}—Fe4—Mg2^{iii}	90.88 (12)
O3^{i}—Fe1—Mg2^{iii}	140.31 (18)	O2—Fe4—Mg2^{iii}	44.45 (10)
O3—Fe1—Mg2^{iii}	92.57 (15)	O7—Si1—O1	110.7 (3)
O1—Fe1—Mg2^{iii}	135.06 (12)	O7—Si1—O5	109.1 (3)
O1^{ii}—Fe1—Mg2^{iii}	42.89 (12)	O1—Si1—O5	110.0 (2)

O2—Fe1—Mg2^{iii}	41.94 (10)	O7—Si1—O6	108.8 (3)
O2^{ii}—Fe1—Mg2^{iii}	87.12 (11)	O1—Si1—O6	109.8 (2)
Mg1^{i}—Fe1—Mg2^{iii}	122.72 (3)	O5—Si1—O6	108.4 (2)
O4^{iv}—Fe2—O4^{v}	93.0 (2)	O7—Si1—Ca4^{iv}	126.9 (2)
O4^{iv}—Fe2—O2^{ii}	86.06 (16)	O1—Si1—Ca4^{iv}	120.97 (16)
O4^{v}—Fe2—O2^{ii}	98.38 (16)	O5—Si1—Ca4^{iv}	64.55 (16)
O4^{iv}—Fe2—O2^{vi}	98.38 (16)	O6—Si1—Ca4^{iv}	44.02 (16)
O4^{v}—Fe2—O2^{vi}	86.06 (16)	O4—Si2—O2	116.1 (2)
O2^{ii}—Fe2—O2^{vi}	173.6 (2)	O4—Si2—O5^{iii}	109.7 (2)
O4^{iv}—Fe2—O1^{vii}	173.16 (17)	O2—Si2—O5^{iii}	108.6 (2)
O4^{v}—Fe2—O1^{vii}	93.61 (16)	O4—Si2—O6	102.0 (2)
O2^{ii}—Fe2—O1^{vii}	91.41 (16)	O2—Si2—O6	109.3 (2)
O2^{vi}—Fe2—O1^{vii}	83.66 (16)	O5^{iii}—Si2—O6	111.1 (2)
O4^{iv}—Fe2—O1	93.61 (16)	O4—Si2—Ca4^{iv}	38.96 (15)
O4^{v}—Fe2—O1	173.16 (17)	O2—Si2—Ca4^{iv}	127.67 (16)
O2^{ii}—Fe2—O1	83.66 (16)	O5^{iii}—Si2—Ca4^{iv}	122.69 (16)
O2^{vi}—Fe2—O1	91.41 (16)	O6—Si2—Ca4^{iv}	63.05 (16)
O1^{vii}—Fe2—O1	79.8 (2)	Si1—O1—Fe1	121.3 (2)
O4^{iv}—Fe2—Ca4^{vi}	95.54 (12)	Si1—O1—Fe3	117.9 (2)
O4^{v}—Fe2—Ca4^{vi}	40.96 (11)	Fe1—O1—Fe3	94.49 (17)
O2^{ii}—Fe2—Ca4^{vi}	139.32 (12)	Si1—O1—Fe2	121.9 (2)
O2^{vi}—Fe2—Ca4^{vi}	45.18 (12)	Fe1—O1—Fe2	96.39 (17)
O1^{vii}—Fe2—Ca4^{vi}	90.52 (11)	Fe3—O1—Fe2	99.16 (17)
O1—Fe2—Ca4^{vi}	136.49 (11)	Si2—O2—Mg2^{iii}	123.8 (2)
O4^{iv}—Fe2—Mg1^{vi}	141.26 (12)	Si2—O2—Fe2^{iii}	123.8 (2)
O4^{v}—Fe2—Mg1^{vi}	87.93 (11)	Mg2^{iii}—O2—Fe2^{iii}	0.0
O2^{ii}—Fe2—Mg1^{vi}	132.13 (12)	Si2—O2—Fe4	125.5 (2)
O2^{vi}—Fe2—Mg1^{vi}	43.01 (12)	Mg2^{iii}—O2—Fe4	90.37 (16)
O1^{vii}—Fe2—Mg1^{vi}	40.72 (10)	Fe2^{iii}—O2—Fe4	90.37 (16)
O1—Fe2—Mg1^{vi}	85.90 (11)	Si2—O2—Fe1	119.7 (2)
Ca4^{vi}—Fe2—Mg1^{vi}	61.31 (3)	Mg2^{iii}—O2—Fe1	95.05 (16)

O3^{i}—Fe3—O3^{vi}	180.0 (3)	Fe2^{iii}—O2—Fe1	95.05 (16)
O3^{i}—Fe3—O1	83.81 (17)	Fe4—O2—Fe1	94.14 (17)
O3^{vi}—Fe3—O1	96.19 (17)	Al3^{iii}—O3—Fe3^{iii}	0.0
O3^{i}—Fe3—O1^{viii}	96.19 (17)	Al3^{iii}—O3—Fe1	97.1 (2)
O3^{vi}—Fe3—O1^{viii}	83.81 (17)	Fe3^{iii}—O3—Fe1	97.1 (2)
O1—Fe3—O1^{viii}	180.0	Al3^{iii}—O3—Fe1^{i}	97.1 (2)
O3^{i}—Fe3—O1^{ix}	83.81 (17)	Fe3^{iii}—O3—Fe1^{i}	97.1 (2)
O3^{vi}—Fe3—O1^{ix}	96.19 (17)	Fe1—O3—Fe1^{i}	97.8 (3)
O1—Fe3—O1^{ix}	98.1 (2)	Al3^{iii}—O3—Mg1^{i}	97.1 (2)
O1^{viii}—Fe3—O1^{ix}	81.9 (2)	Fe3^{iii}—O3—Mg1^{i}	97.1 (2)
O3^{i}—Fe3—O1^{vii}	96.19 (17)	Fe1—O3—Mg1^{i}	97.8
O3^{vi}—Fe3—O1^{vii}	83.81 (17)	Fe1^{i}—O3—Mg1^{i}	0.00 (5)
O1—Fe3—O1^{vii}	81.9 (2)	Al3^{iii}—O3—H3	109 (7)
O1^{viii}—Fe3—O1^{vii}	98.1 (2)	Fe3^{iii}—O3—H3	109 (7)
O1^{ix}—Fe3—O1^{vii}	180.0 (3)	Fe1—O3—H3	125 (3)
O3^{i}—Fe3—Mg1^{viii}	138.39 (13)	Fe1^{i}—O3—H3	125 (3)
O3^{vi}—Fe3—Mg1^{viii}	41.61 (13)	Mg1^{i}—O3—H3	125 (3)
O1—Fe3—Mg1^{viii}	137.79 (11)	Si2—O4—Ca4^{iv}	110.8 (2)
O1^{viii}—Fe3—Mg1^{viii}	42.21 (11)	Si2—O4—Fe4^{iv}	110.8 (2)
O1^{ix}—Fe3—Mg1^{viii}	88.81 (11)	Ca4^{iv}—O4—Fe4^{iv}	0.0
O1^{vii}—Fe3—Mg1^{viii}	91.19 (11)	Si2—O4—Fe2^{iv}	143.0 (2)
O3^{i}—Fe3—Mg1^{vi}	138.39 (13)	Ca4^{iv}—O4—Fe2^{iv}	96.86 (18)
O3^{vi}—Fe3—Mg1^{vi}	41.61 (13)	Fe4^{iv}—O4—Fe2^{iv}	96.86 (18)
O1—Fe3—Mg1^{vi}	88.81 (11)	Si2—O4—Mg2^{iv}	143.0 (2)
O1^{viii}—Fe3—Mg1^{vi}	91.19 (11)	Ca4^{iv}—O4—Mg2^{iv}	96.86 (18)
O1^{ix}—Fe3—Mg1^{vi}	137.79 (11)	Fe4^{iv}—O4—Mg2^{iv}	96.86 (18)
O1^{vii}—Fe3—Mg1^{vi}	42.21 (11)	Fe2^{iv}—O4—Mg2^{iv}	0.00 (6)
Mg1^{viii}—Fe3—Mg1^{vi}	60.55 (4)	Si1—O5—Si2^{vi}	141.0 (3)
O3^{i}—Fe3—Mg1^{i}	41.61 (13)	Si1—O6—Si2	140.8 (3)
O3^{vi}—Fe3—Mg1^{i}	138.39 (13)	Si1—O6—Ca4^{iv}	110.9 (2)
O1—Fe3—Mg1^{i}	91.19 (11)	Si2—O6—Ca4^{iv}	83.66 (18)

O1^{viii}—Fe3—Mg1^{i}	88.81 (11)	Si1—O7—Si1^{ix}	143.4 (4)
O1^{ix}—Fe3—Mg1^{i}	42.21 (11)		

Symmetry code(s): (i) −*x*, −*y*, −*z*+1; (ii) −*x*, *y*, −*z*+1; (iii) *x*, *y*, *z*+1; (iv) −*x*+1/2, −*y*+1/2, −*z*+1; (v) *x*−1/2, −*y*+1/2, *z*−1; (vi) *x*, *y*, *z*−1; (vii) −*x*, *y*, −*z*; (viii) −*x*, −*y*, −*z*; (ix) *x*, −*y*, *z*; (x) *x*−1/2, −*y*+1/2, *z*.

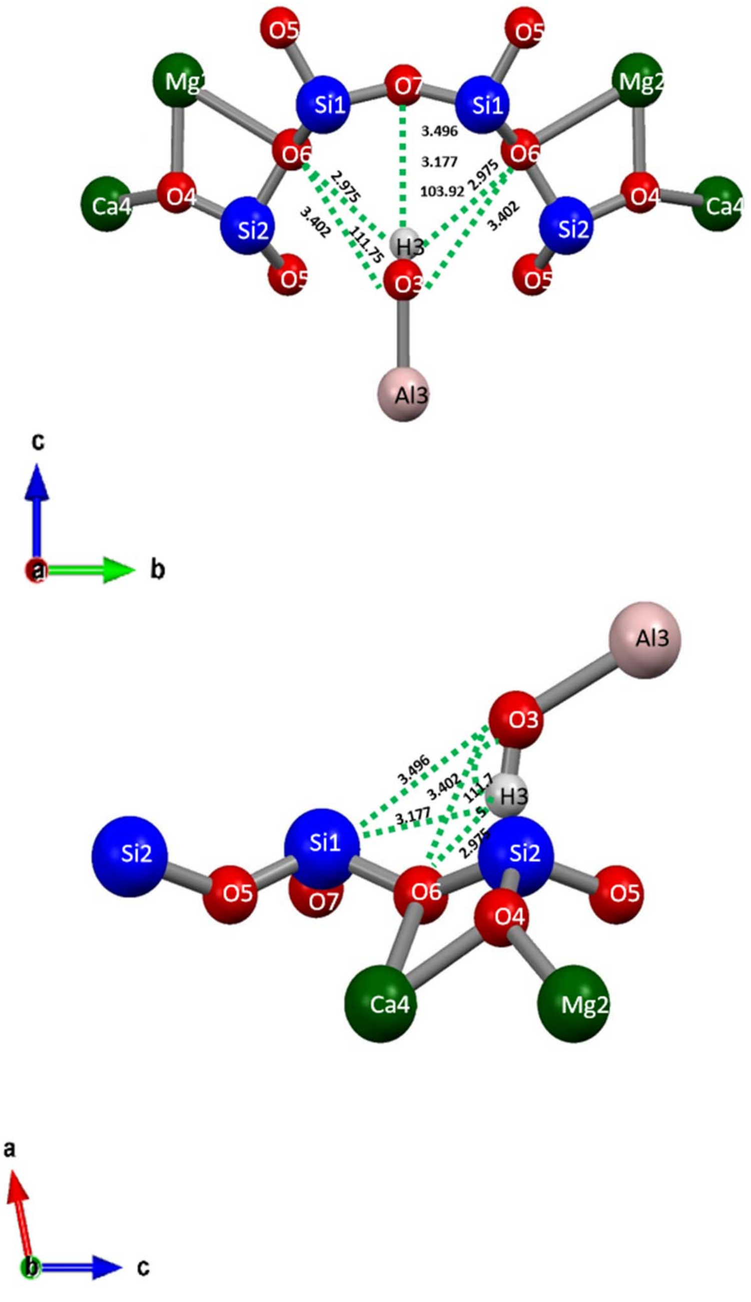

Figure S5 A view along *a* (a) and along *b* (b) showing three weak H-bonds involving O(6) and O(7) atoms as acceptor. A PLAT420_ALERT_2_B alert has been generated during the CIF validation

process of the two refined structures (fibre_T and fibre_C), advising that a D-H bond without acceptor would be present.

However, similarly to what has been considered for kaersutite, a Ti-rich calcium amphibole (Gatta *et al.*, 2016), we observe three potential weak H-bonds with O(6) × 2 and O(7) as acceptors (*i.e.*, H···O(6) = 2.975 Å, O(3)···O(6) = 3.402 Å and O(3)–H···O(6) = 111.75°; H···O(7) = 3.177 Å, O(3)···O(7) = 3.496 Å and O(3)– H···O(7) = 103.9°). Distances and angles have been calculated using Mercury software.
